# Assessment of ecosystem services of an urbanized tropical estuary with a focus on habitats and scenarios

**DOI:** 10.1371/journal.pone.0203927

**Published:** 2018-10-05

**Authors:** Carlos Zapata, Araceli Puente, Andrés García, Javier Garcia-Alba, Jorge Espinoza

**Affiliations:** 1 Department of Hydrography, Instituto Oceanográfico de la Armada (INOCAR), Guayaquil, Guayas, Ecuador; 2 Enviromental Hydraulics Institute, Universidad de Cantabria, Santander, Cantabria, Spain; Universidade de Aveiro, PORTUGAL

## Abstract

Tropical estuaries are one of the most valuable ecosystems on the planet because of the number of ecosystem services they provide. The increasing anthropogenic pressure to which these estuaries are subject has caused a reduction in their natural capital stock. Therefore, the application of a pragmatic and rational ecosystem-based management approach to sustainably manage the multiple ecosystem services provided by this ecosystem is necessary. The aim of our study is to present an approach that combines prospective scenarios with habitat-based perspective to assess the supply capacity of ecosystem services, plus determine the impact of protected areas in an urbanized tropical estuary. The current situation and two scenarios were generated to evaluate the capacity of habitats to supply ecosystem services. This type of assessment will allow the decision makers to visualize the effect of their choices or the occurrence of events which might produce significant changes in the estuary. Thus, over time, measures can be taken to sustain the supply of ecosystem services. We determined that the establishment of protected areas have a positive impact; however, the effect is not the same for all of them. Consequently, indicating that actions such as community participation, research, education, management planning and infrastructure development must accompany the development of a protected area.

## Introduction

Estuaries are complex transitional environments [1] as they are the interface between ocean and river environments and therefore marine and terrestrial systems. As a result, they provide a significant variety of physical, chemical and geomorphologic environmental conditions [2,3]. In tropical regions, estuaries house important habitats such as mangrove forests and seagrass beds [4], which means they are among the most productive and valuable ecosystems on the planet due to the quantity and quality of ecosystem services (ESs) which they supply [5,6].

However, their capacity to supply well-being to humans has generated adverse effects. This comes as a result of the growing pressure yielded by factors such as population growth, urbanization [7], expansion of aquaculture [8], industry and agriculture [9]. On many occasions, the pressure has grown to a point where it has generated conflicts among different groups of interest. Each attempting to maximize the benefits to be obtained from nature [10]. This situation has produced governance complications and caused the deterioration and decrease of the natural capital stock available [11].

The deterioration of this ecosystem requires that all stakeholders take on a pragmatic and rational approach to ecosystem-based management (EBM) maintaining the health of the ecosystem (structure and function) and the ecosystem services demanded by the society [12]. The establishment of protected areas (PAs) is one of the tools that the EBM has come up with for long-term maintenance of ecosystems capacity [13,14,15]. The creation of PAs alleviates the pressure on the tropical estuaries [14] and helps to provide a better insight into their natural relationships [13]. Another tool that is available under the focus of EBM is scenario building. This tool is used to explore the consequences of expected changes on natural resources and ecosystem services [15,16] under different management options; this information is relevant when making decisions [17].

Scenarios are visualizations of possible future events resulting from a combination of trends and policies [18] There are several methodologies for scenario construction [19], which can be classified into three schools of thought: intuitive logic, the French school (prospective), and probabilistic modified trends [20]. The prospective school proposes that scenarios can serve not just as a visualization of possible futures but also as an orientation to build an idealized future through the scenario building and strategic planning [21,22]. Therefore scenarios can serve as a guide to policymakers and provide a basis for future actions [20]. The prospective scenarios are more often used in public sector planning [20,23]. For this reason, in several Latin American countries, it has been widely used in strategic planning processes. [24].

Ecosystem assessment is a systematic process aiming at providing support for decision making relating to issues of ecosystem services and sustainable development; the habitat-perspective is one of the most common analytical strategies used. It is based on stock and condition of habitat, ecotopes or biomes, etc. [25]. The aim of our study is to present an approach that combines prospective scenarios with habitat-based perspective to assess the supply capacity of ecosystem services, plus determine the impact of protected areas in an urbanized tropical estuary.

This approach will allow us to carry out a swift and general assessment, by means of expert judgment, of the supply capacity of the ecosystem services provided by the habitats in the study area. The prospective scenarios will aid in highlighting variations in the supply capacity of ecosystem services as a consequence of a particular choice, policy decision or event occurrence.

## Methodology and results

### Study area

**Fig 1:**
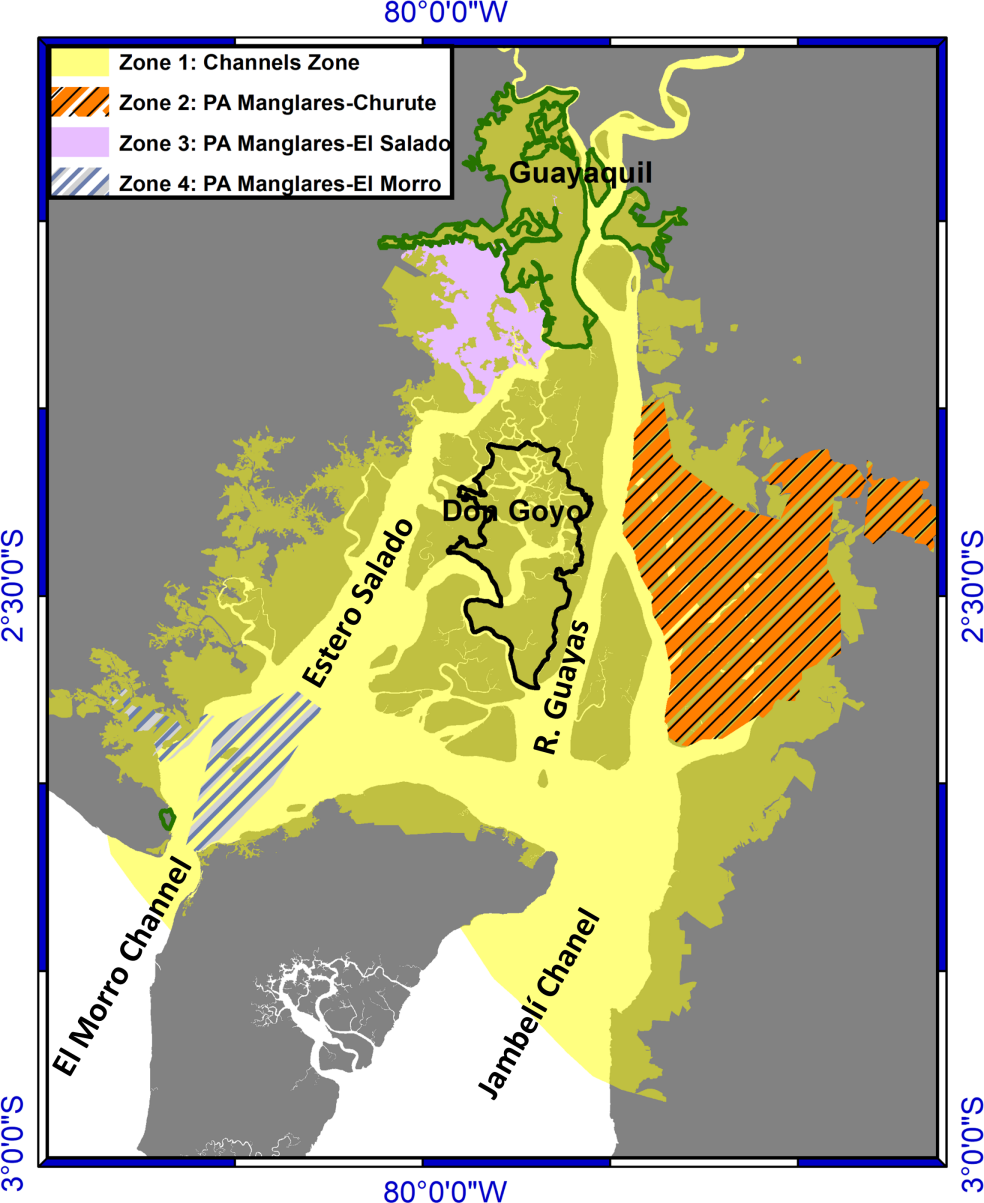
Study area and the four zones.

The study area (SA) is located in an estuarine system in the Gulf of Guayaquil [26] that includes the estuary of the Guayas River, the Estero Salado and the Churute and Taura Rivers [27]. The SA covers an area of 3721 km^2^, and it was delimited based on hydrodynamic (S1 Video), salinity (S1 Dataset), conservation criteria, and the typical uses of the estuarine water. In the North, the city limit of Guayaquil was employed; in the South, El Morro channel in the Estero Salado, and the boundary of the polyhaline zone of the Jambelí channel in the Guayas River were used; in addition, the intertidal and supratidal zones, populated areas and ports, mangrove forests and the shrimp pools were considered for defining the Lateral Limits (Fig 1).

Guayaquil is the most populated city with the highest population growth rate in Ecuador [28]. Its rapid expansion and slow growth of the capacity of wastewater treatment plants (especially with secondary treatment) has converted the city into a source of pollution from industrial discharges, domestic sewage [29–31] and oil hydrocarbon residuals [32]. The port of this city has been of most importance to Ecuador since 1963 and, together with the port terminals, located both in the Guayas River and in the Estero Salado, they manage approximately 80% of the non-oil dealer cargo nationally [33]. Since 1969, the shrimping industry has swelled throughout the study area, causing a 24% loss of mangrove coverage [34–36]. In an effort to conserve this habitat, protected areas and protected species were established [37], as well as the sustainable development of community-held areas[38,39].

**Fig 2:**
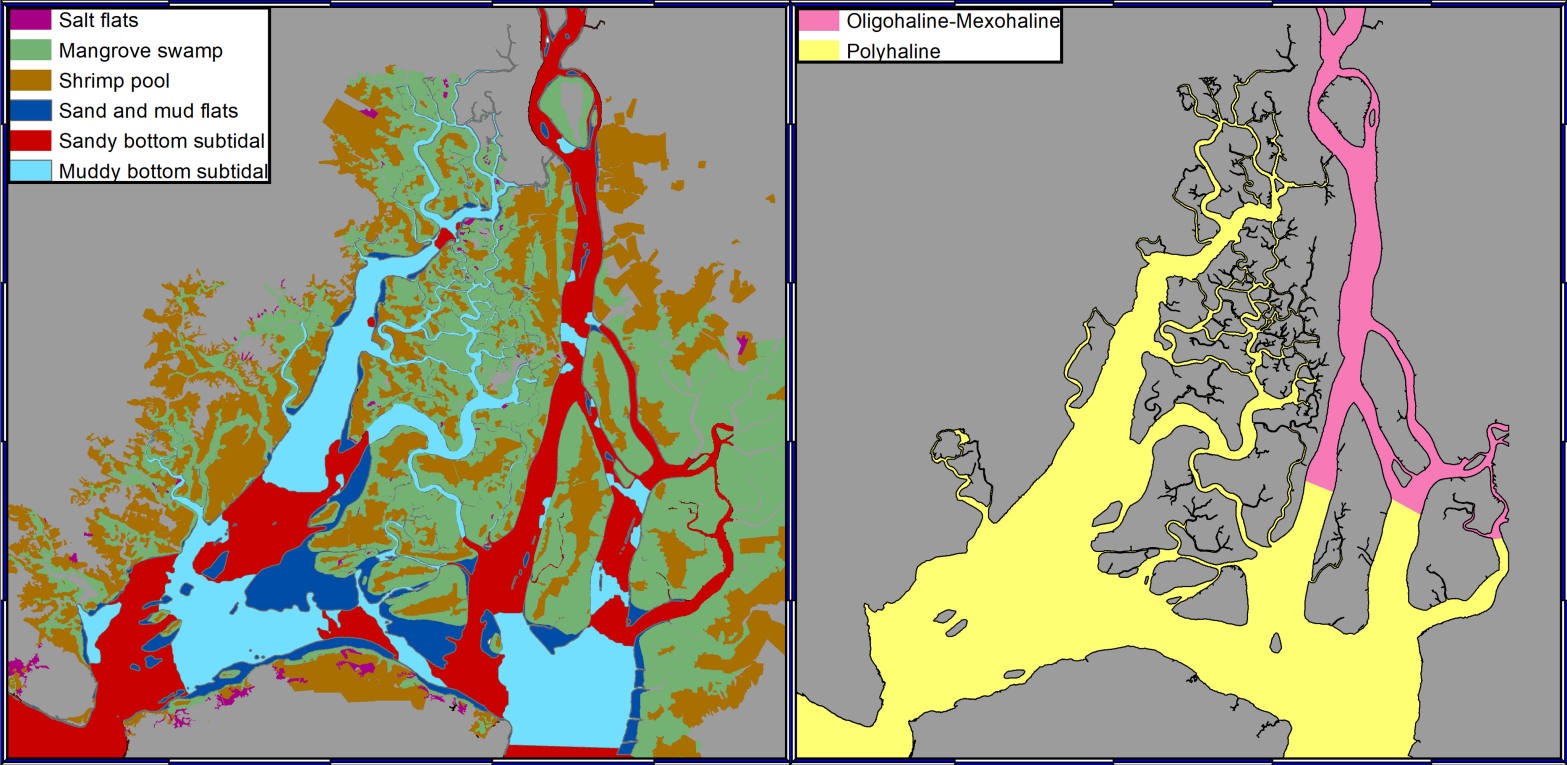
Habitat distribution within the study area.

The study area was subdivided into four zones based on hydrodynamic and environmental criteria (Fig 1). Zone 1 includes an area that sits outside of the other zones, which we call the “Channels Zone”. Zone 2 includes the PA “Churute Mangroves”. Zone 3 contains PA “El Salado Mangroves”. Zone 4 encompasses the PA “El Morro Mangroves.” Since there is no official classification of habitats within the SA, one was accomplished by taking into consideration the classification of habitats in similar estuarine systems. The following natural habitats were defined [40]: salt flats, sand and mudflats, mangrove swamp, sandy-bottom subtidal, muddy-bottom subtidal, oligo-mesohaline water column, and polyhaline water column. Additionally, the shrimp pools were included as a manmade habitat [41,42]. The spatial distribution of the habitats is shown in Fig 2 (S1 Dataset).

### Assessment of the capacity of supply of ecosystem services

**Fig 3:**
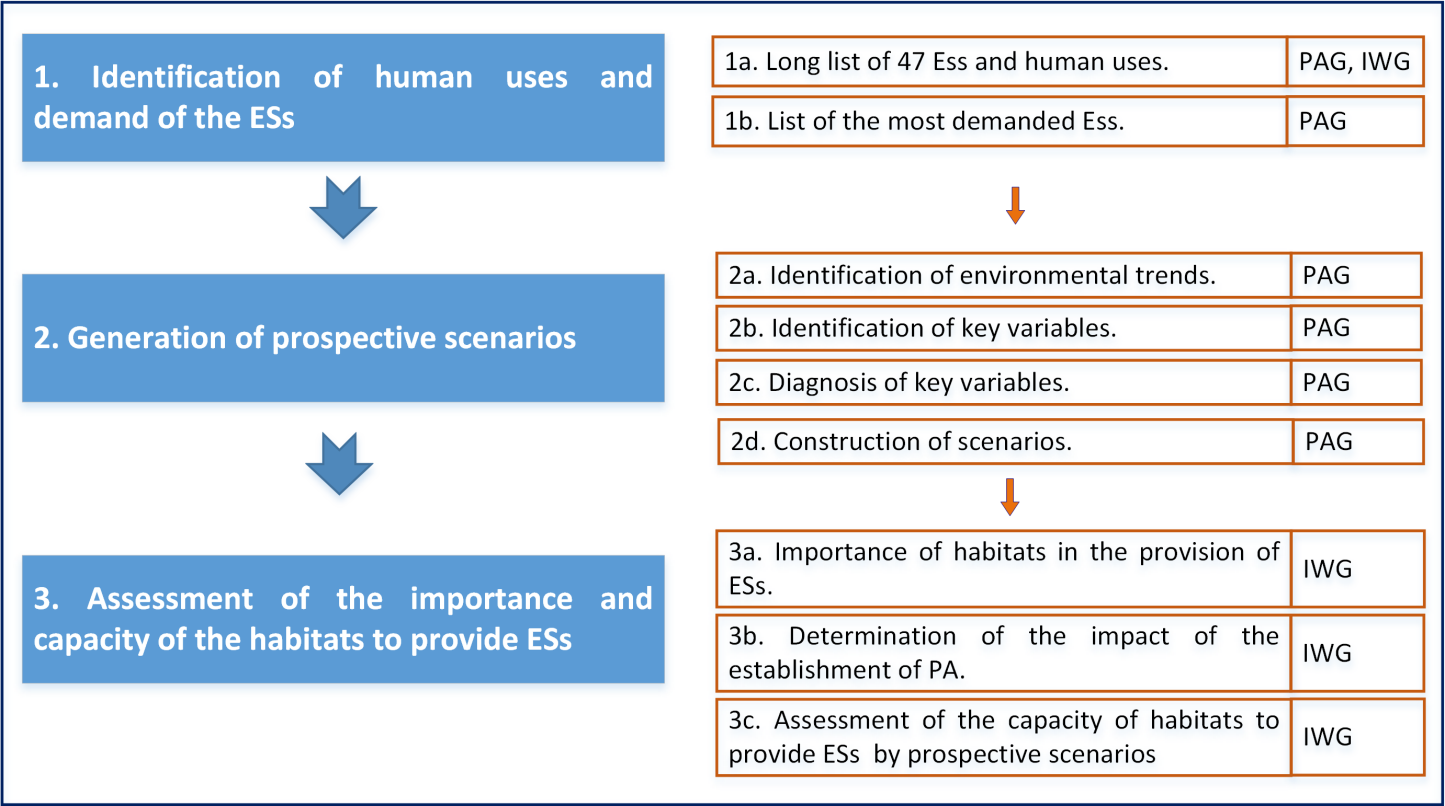
Ecosystem services assessment framework.

The approach that was used to assess the availability of the ecosystem services of an urbanized tropical estuary involved three stages (Fig 3): 1) identification of human uses and demand of the ESs; 2) generation of prospective scenarios; and 3) assessment of the importance and capacity of the habitats in order to provide ESs by prospective scenarios.

For the construction of the prospective scenarios and the valuation of ecosystem services, two working groups were created:

- The Intersectoral Working Group (IWG): to build up this group, letters of invitation were sent to those responsible for the environmental area of the local, provincial, and municipal institutions related with the governance of the estuary. In addtion, invitations were sent to university professors and researchers in the field of chemistry and biology who carried out projects in the study area, port managers, and ecosystem services users. Three workshops were carried out (December 2016, June 2017, July 2017). At the beginning of each workshop, a presentation of the objectives, agenda, and information related to the ESs was presented to the participants; allowing to attain the verbal consent from participants. This group partook in stage 1 and stage 3. Invitations were addressed both to the state institutions and civil society; however, the assistance was mostly from governmental institutions and the academy. Table 1 shows the list of the stakeholders and their affiliations.

**Table 1:** Affiliation of IWG members.

Stakeholder affiliation	Number
Environmental Ministry	02
Tourism Ministry	01
Guayaquil Port Authority	01
Municipality of Guayaquil	01
Prefecture of Guayas province	01
Oceanographic Institute of the Navy (INOCAR)	02
Estatal University	01
Agraria University	01
Aquaculture sector	01

- The Prospective Analysis Group (PAG): This group was made up of one shrimp farm manager and three experts who have participated in various projects related to the Gulf of Guayaquil. An expert in prospective advised the PAG on the development of the scenarios. This group was in charge of presenting a list of ecosystem services and a list of human uses, assessing the ESs demand for stage 1, and producing the scenarios in stage 2. Table 2 shows the affiliation and the field of knowledge of each member of this group.

**Table 2:** Affiliation of PAG members.

Affiliation	Field of knowledge
INOCAR	Hydrography, navigation, and ecosystem services
Polytechnic College of the Littoral	Coastal processes and environment
Estatal University/INOCAR	Chemical Process
Shrimp sector	Shrimp farm manager

### Identification of human uses and the demand of ecosystem services

The identification and description of the system were finalized based on its thematic delimitation [43]. In this case, the human uses give us a complete visualization of the situation within the study area. To define the human uses within an estuarine system, the PAG used two bibliographic sources: 1) List of human uses for the entire coastal zone of Ecuador defined by the Secretary of Planning of Ecuador, through a series of workshops—that is published in the ‘Plan for the Coastal Marine Area’ [28] and 2) List of human uses defined by the TIDE project for four North-West EU estuaries [44]. The PAG, based on its experience, selected 15 human uses that coincided with both lists, 11 uses from TIDE list and 03 human uses from SENPLADES list. This was carried out in order to describe the estuarine system in a better way. Subsequently, the 29 human uses were grouped into 11 sectors (S1 Table) based on their similarities and mapped for a better understanding of the system.

During the first workshop, the IWG and PAG, through a participative process [45], determined the most-demanded ecosystem services for each human use using the following steps:

- Definition of a general list of the ESs. The IWG reviewed the list of services elaborated in the TIDE project for industrialized estuaries [46,47]. The ESs are grouped into four categories: cultural, provisioning, regulating, and supporting. The definition of some services were broadened, and two provision services were included: 1) water for aquaculture use and 2) fossil energy sources (S2 Table).
- Determination of the main ecosystem services. The PAG completed a qualitative assessment of the demand of ecosystem services for human use, using a scale from 0 to 3 (where 0: not demanded; 1: little demand; 2: moderately demanded; and 3: highly in demand). The final scoring was determined by applying the criteria of the mode [48], to the assessment that each member in the group did. Afterward, the ecosystem services that scored 3 were chosen for each human use (S3 Table).

Consequently, from the survey of the ESs demand by human uses, 31 ESs were selected: 1 supporting, 7 provisioning, 18 regulating and 5 cultural. The services with the highest demand were biodiversity, information for cognitive development, and regulation of water quality (including transport of pollutants and nutrient excess, navigation, and regulation of sedimentation and erosion in the bodies of water). Likewise, the human use with the highest demand of ESs was soil for aquaculture.

### Generation of prospective scenarios

The National Center for Strategic Planning (NCSP) of Perú developed a methodology for strategic planning based on the MEYEP prospective method. It has two major phases, prospective analysis and strategic. The prospective analysis phase has five stages: 1) Analysis and understanding of the research topic; 2) Identification and analysis of the trends; 3) Key variable selection; 4) Diagnosis of key variables; 5) Construction of scenarios. In this approach, the analysis and understanding of the research topic were carried out during step 1a through the identification and analysis of human uses which allowed us to understand the demands of ecosystem services within the study area. The other stages of NCSP methodology were carried out during the second stage of our approach.

#### a. Identification of environmental trends

After analyzing the system, it was necessary to evaluate how the environmental fluctuations could impact it. This was completed by identifying the behavioral trends of the environment, that could influence our system in the future and be able to analyze their impact [49]. Global trends defined by the United Nations for Latin America were considered for this analysis. These trends have a temporal scope year of 2030 [50], the same as in this study. For the selection of the trends that affect the system, the degree of relationship that the trend has with the estuarine system (relevance) and the reliability of the qualitative or quantitative data that support the trend (evidence) were considered. These two criteria were scored on a scale of 1–5 by the PAG [43]. Because of this assessment, the two highest-scoring trends were selected: 1) climate change and 2) citizen empowerment and government transformation.

#### b. Identification of key variables

A condition of change was added to both the environmental trends (2) and the human uses (29) to transform them into variables. These were conceptually defined, and one or two indicators were established. These indicators allowed the characterization of their current situation and evolution over time to determine trends or future disruptive events [51]. In this stage, 31 variables were defined (S1 Table).

**Fig 4:**
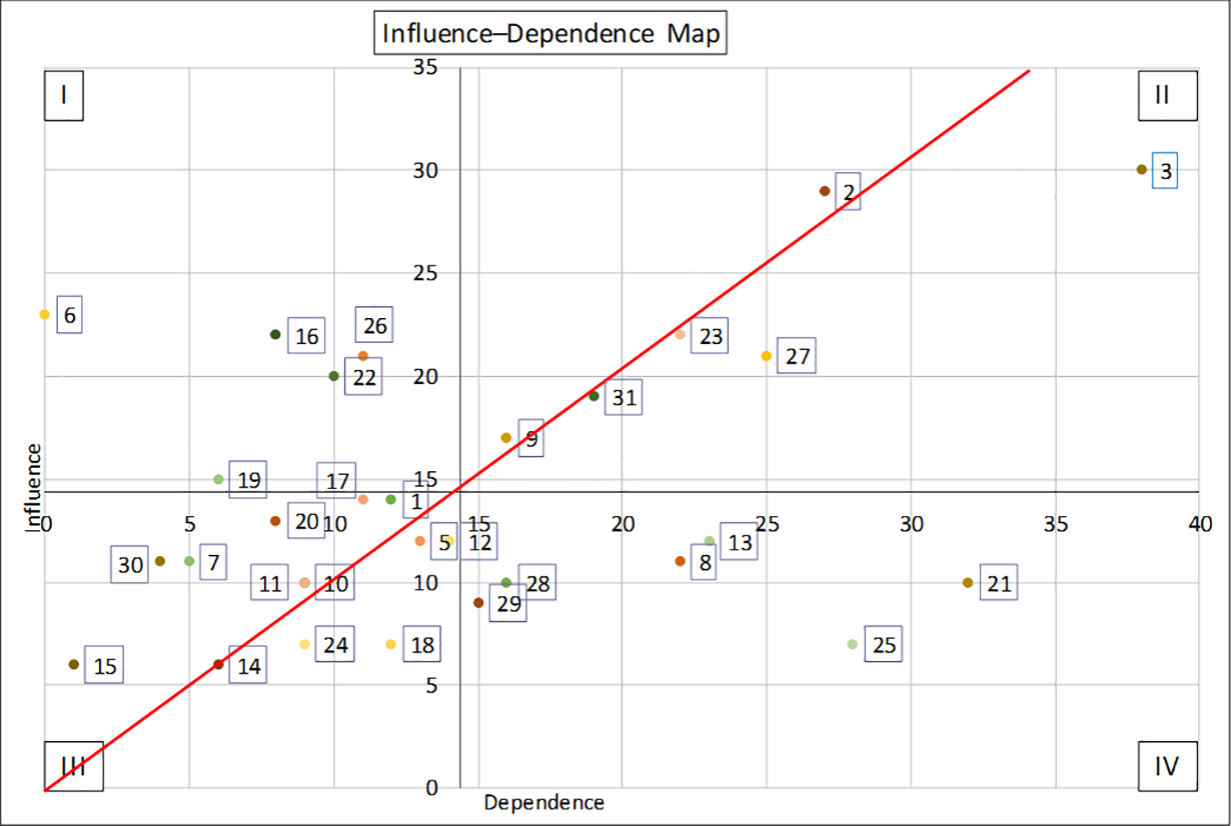
Influence-dependence map with the location of each variable in the different quadrants.

The next step involved the construction of a Structural Matrix. We used only direct relationships to assess how the variable (i) influenced the variable (j) [52,53]. The scores varied from 0 to 3 (0: no influence, 1: weak influence, 2: moderate influence, 3: strong influence). The sum of the rows provides the influence, whereas the sum of the columns represents the dependence of each variable (S4 Table). Depending on the result obtained in the Structural Matrix, each one of the variables was graphed on the influence–dependence plane (Fig 4). This plane shows a cloud of points located around the diagonal line, which indicates that it is an unstable system. Thus, any one action on any one variable affects other variables, as well as itself [21,54].

In this prospective view, different authors recommend the selection of key variables from quadrant II (strong influence and strong dependence), but at the same time, they agree that nothing could replace the opinions of experts and analysts. This means that to achieve a better description of the study system, the possibility of selecting key variables from quadrant IV is viable [21,43,49]. In this case, the PAG chose five variables from quadrant II and one from quadrant IV.

#### c. Diagnosis of key variables

**Fig 5:**
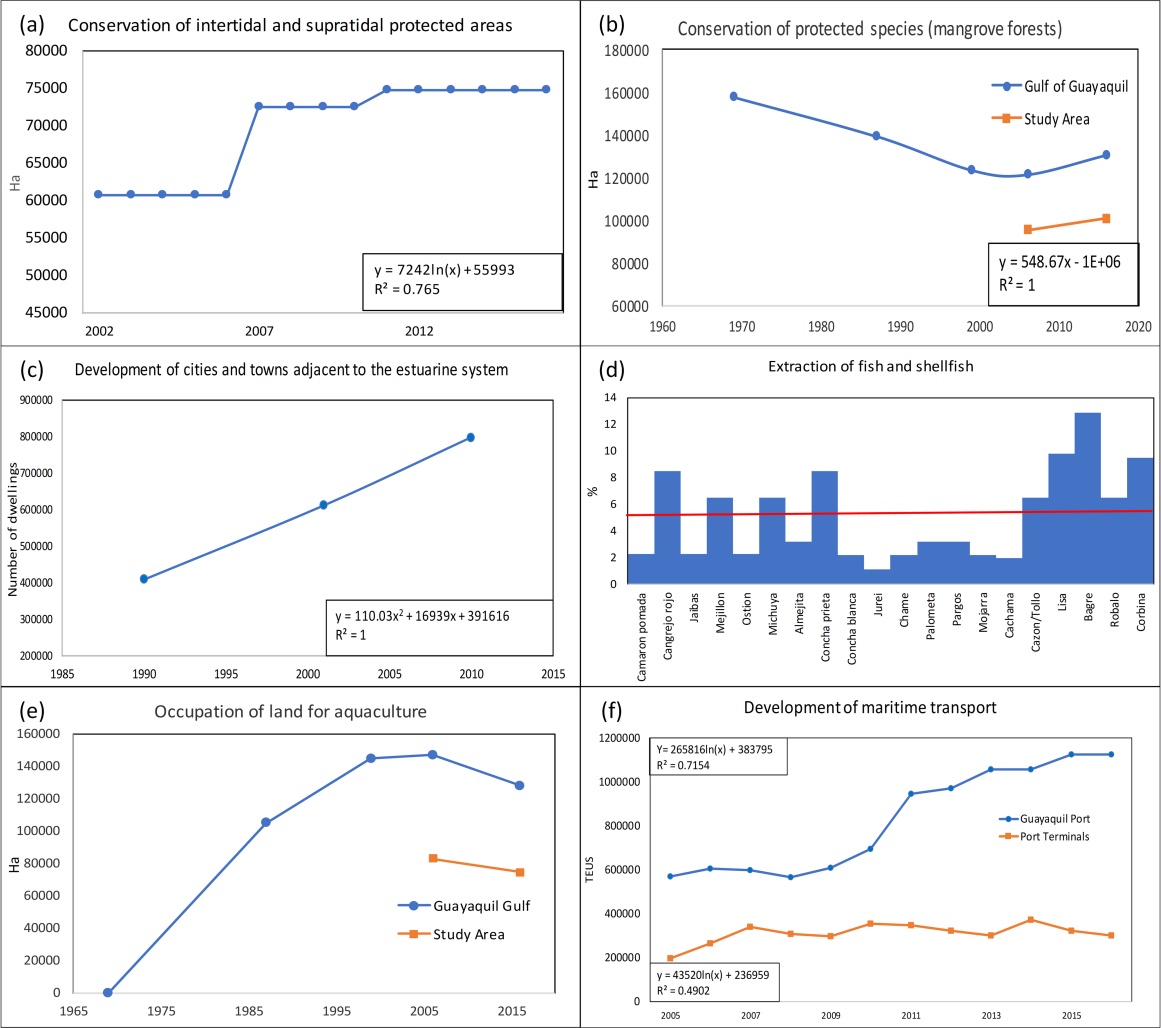
Historical trends of the key variables. (a) PA has a logarithmic growth. (b) In blue, the coverage of the mangrove forests in the Gulf of Guayaquil, and in orange the trend within the SA which has linear growth. (c) Shows a polynomial trend of growth of the homes within the SA. (d) A survey carried out on fishermen shows the percentage reduction of fishing of different native species [55], the red line represents the average of the expressed percentages. (e) In recent years, a decrease of shrimp farms has occurred; the 2016 data reflects the shrimp farms extension from the last legalization census. It is believed that this extension will remain constant in the future because the current legal regulations prohibit new permits for shrimp farms. (f) Cargo growth in both the Port of Guayaquil and the private port terminals show a logarithmic growth, even though the number of cargo ships that sail within the SA has decreased, which indicates that the size of the vessels has increased.

The six key variables were analyzed through their indicators to establish their current values, historical behavioral patterns, and their projected values up to the year 2030. For the quantitative variables to calculate both the current situation and the projected value in 2030, the most appropriate formula for projection was selected and applied. For the qualitative variables or those that do not have a data series either recovered or collected over time, the trends were obtained through questions to experts or key actors [43,49], (Fig 5).

In Table 3, the current situation, and the projected situation for 2030 are shown. With a goal of neither overestimating nor underestimating the variables, the future values were validated by experts in each of the areas. This information allows us to construct a trend-based scenario [49].

**Table 3:** Key variables of the system.

Var.	Key variable	Current situation	Future value
2	Conservation of intertidal and supratidal protected areas	74727 (Ha)	80379 (Ha)
3	Conservation of protected species (mangrove forests)	95433 (Ha)	108600(Ha)
	Development of cities and towns adjacent to the estuarine system	928701	1271075
21	Extraction of fish and shellfish	-5%	-6%
27	Occupation of land for aquaculture	74555 (Ha)	74555 (Ha)
9	Development of maritime transport	1451353(TEUS)	1603840(TEUS)

A causal analysis was completed for each of the key variables using the Structural Analysis Matrix and analyzing the chains of influences that each key variable has in connection with the rest of the variables [43]. It was determined that a strong influence exists in both ways among five of the six key variables as well as the variables related to the discharge of sewage water, industrial waste, and shrimp pools. Because data regarding the quantity of each type of discharge is not available, these variables were grouped into one. To represent their variation within the scenarios, the concentration limits of chemical parameters of the estuarine waters as determined in the national environmental legislation were considered. The causal analysis can be found in S1 Fig.

#### d. Construction of prospective scenarios

With the information generated in the preceding steps, three types of prospective scenarios could be constructed optimal, trend-based and exploratory [43,49]. For our study, the PAG developed just two scenarios, the trend-based scenario, and an exploratory scenario:

- The trend-based scenario follows the logic of what could occur if nothing is done and if historical patterns of the key variables are kept without distortions. This scenario is considered to be the most likely [49].
- The exploratory scenarios are constructed with the inclusion of disruptive events in the system, whose occurrence can provoke changes in the historical behavior of the key variables. It is possible to build as many exploratory scenarios as disruptive events can be defined or combined [49]. In this study, only one exploratory scenario was created which considered the occurrence of a disruptive event “Reduction of the environmental control measures”. The effects that the disruptive event (positive, negative, or neutral) had on each key variable were analyzed.

Table 4 summarizes the two scenarios developed by the PAG. The IWG validated them during the second workshop.

**Table 4:** Trend-based and exploratory scenarios.

Trend-based scenario	Exploratory scenario
14% increase of protected areas extension in the wetland area of ¨Don Goyo¨	No new protected areas were established.
8% increase in mangrove forest areas due to reforestation of abandoned shrimp pools	A 10% decrease in mangrove forests, mainly outside of protected areas, occurs.
The city of Guayaquil is projected to grow towards the southwest, thus putting pressure on mangrove forest areas located in the western margin of the Estero Salado.	The growth pressure of the city of Guayaquil causes a decrease in the area of mangrove forests outside of protected areas and within the protected area of ¨El Salado¨.
A 5% decrease in fishing is projected.	A 6% decrease in fishing is projected.
The transported cargo via maritime routes grows by 11%, the dredging of the main navigation routes is maintained, and the presence of invasive species has increased.	The transported cargo via maritime routes grows by 11%, and the dredging of main navigation routes is maintained, and the presence of invasive species has increased.
The shrimp pool area is maintained. The shrimp production increases due to technological advancements and use of supplemental feeds; the discharge from the shrimp pools increases, thus producing an increment in the pollution of the estuarine system.	The area of shrimping pools increases to an area slightly larger than in 2006. The shrimp production increases due to technological advancements and use of supplemental feeds; the discharge from the shrimp pools increases, thus producing an increment in the pollution of the estuarine system.
The pollution coming from the sewage of the cities and industry has increased even though a new treatment plant was opened in Guayaquil.	The pollution coming from the sewage of the cities and industry has increased even though a new treatment plant was opened in Guayaquil.

### Assessment of the importance and capacity of the habitats to provide ESs, by prospective scenarios

#### a. Importance of habitats in the provision of ecosystem services

To determine the importance that each habitat has in the provision of the ESs, during the second workshop, the IWG answered the following question: ¨The habitat… has… in the provision of the ecosystem system…? ¨ [46,47]. Each IWG member assigned a score between 1 and 5 (1: no importance, 2: very low importance, 3: moderate importance, 4: importance, 5: essential importance). A final score was determined by applying the mode criteria [48] to the assessments that the IWG members completed. Throughout this workshop, the PAG gave a presentation to the IWG about the methodology that was used for the formation of the current situation, trend-based, and exploratory scenarios to validate them.

The results of this assessment are shown in Table 5. The mangrove swamp is the habitat that reached the highest score globally and also in each one of the ecosystem services categories. The water column habitats have essential importance in the provision of water for aquaculture, navigation, regulation of water quantity and quality in the estuary, opportunities for recreation and information for cognitive development. The sand and mudflat habitats have importance for biodiversity, food, and regulation of extreme events or disturbances.

**Table 5:** Assessment of the importance that each habitat has in the provision of different types of SE. AQR: Air quality regulation; CR: Climate regulation; REE: Regulation of extreme events or disturbance; WQR: Water quantity regulation; WQlR: Water quality regulation.

		1		2	3	4	5	6	7	8		9	10	11	12	13	14	15	16	17	18	19	20	21	2	23	24	25	26		27	28	29	30	31	
		A		P	P	P	P	P	P	P		R	R	R	R	R	R	R	R	R	R	R	R	R	R	R	R	R	R		C	C	C	C	C	
	Supporting	Biodiversity	Provisioning	Food: Animals	Water for industrial uses	Water for aquaculture	Water for energy use	Water for navigation	Raw materials: Renewable soil materials: sand materials: sand	Raw materials: Platform	Regulating	AQR: Removing harmful particles	AQR: Air-water exchange	CR: Carbon sequestration and burial	CR: Heat exchange regulation	REE: Flood water storage	REE: Peak discharge buffering	REE: Water current reduction	REE: Wave reduction	WQR: Drainage of river water	WQR: Prevention of saline intrusion.	WQR: Dissipation of tidal and river energy energy	WQR: Landscape maintenance	WQlR: Transport of pollutants and excess nutrients	WQlR: Reduction of excess loads coming from the catchment	Erosion and sedimentation regulation by water bodies	Erosion and sedimentation regulation by biological mediation	Prevention of establishment of harmful invasive species	Pollination	Cultural	Aesthetic information	Opportunities for recreation & tourism	Inspiration for culture, art, and design	Spiritual experience	Information for cognitive development	General
Salt flats	2	2	2	2	1	1	1	1	1	5	2	2	1	1	2	3	3	3	2	2	1	1	4	1	2	4	1	2	2	3	2	3	2	2	5	2
Mangrove swamp	5	5	3	5	2	3	1	1	4	5	4	5	5	5	5	5	5	5	5	3	1	2	5	1	5	5	5	5	5	5	5	5	5	5	5	4
Shrimp pool	2	2	2	5	1	1	1	1	3	2	2	1	3	1	2	3	3	3	2	4	1	1	2	2	2	2	1	1	1	2	2	2	1	1	5	2
Sand and mudflats	4	4	2	4	1	1	1	1	2	3	3	1	1	1	2	5	4	4	3	4	1	2	3	1	2	3	3	3	1	4	4	4	3	3	5	3
Sandy bottoms subtidal	2	2	1	2	1	1	1	1	2	2	2	1	1	2	2	1	1	1	1	5	1	4	2	1	2	2	2	3	1	3	2	2	2	2	5	2
Muddy bottoms subtidal	3	3	2	3	1	1	1	1	2	2	2	1	1	2	2	1	1	1	1	5	1	4	2	1	2	2	2	3	1	2	2	2	1	1	5	2
Oligo-Mesohaline water column	3	3	3	3	3	5	4	5	3	1	3	1	4	1	2	1	1	1	1	5	5	5	4	5	5	4	1	3	1	3	1	5	3	1	5	3
Polyhaline water column	3	3	3	3	4	5	3	5	3	1	3	1	4	1	2	1	1	1	1	5	5	5	4	5	5	4	1	3	1	3	1	5	3	1	5	3

The salt flats, sandy- and muddy-bottom and shrimp pool habitats were assessed as very low importance in the general assessment of the ESs. It is important to note that the shrimp pool habitat was man-made strictly for breeding purposes of one species. Despite its low scores, in this assessment, its extension has increased over time because it is one of the main sources of income and employment in the country [56]. The expansion of this industry has caused a decrease of almost 96% in the salt flats habitat and nearly 24% in the mangrove swamp habitat, which has produced a reduction in the provision of ecosystem services in these habitats.

**Fig 6:**
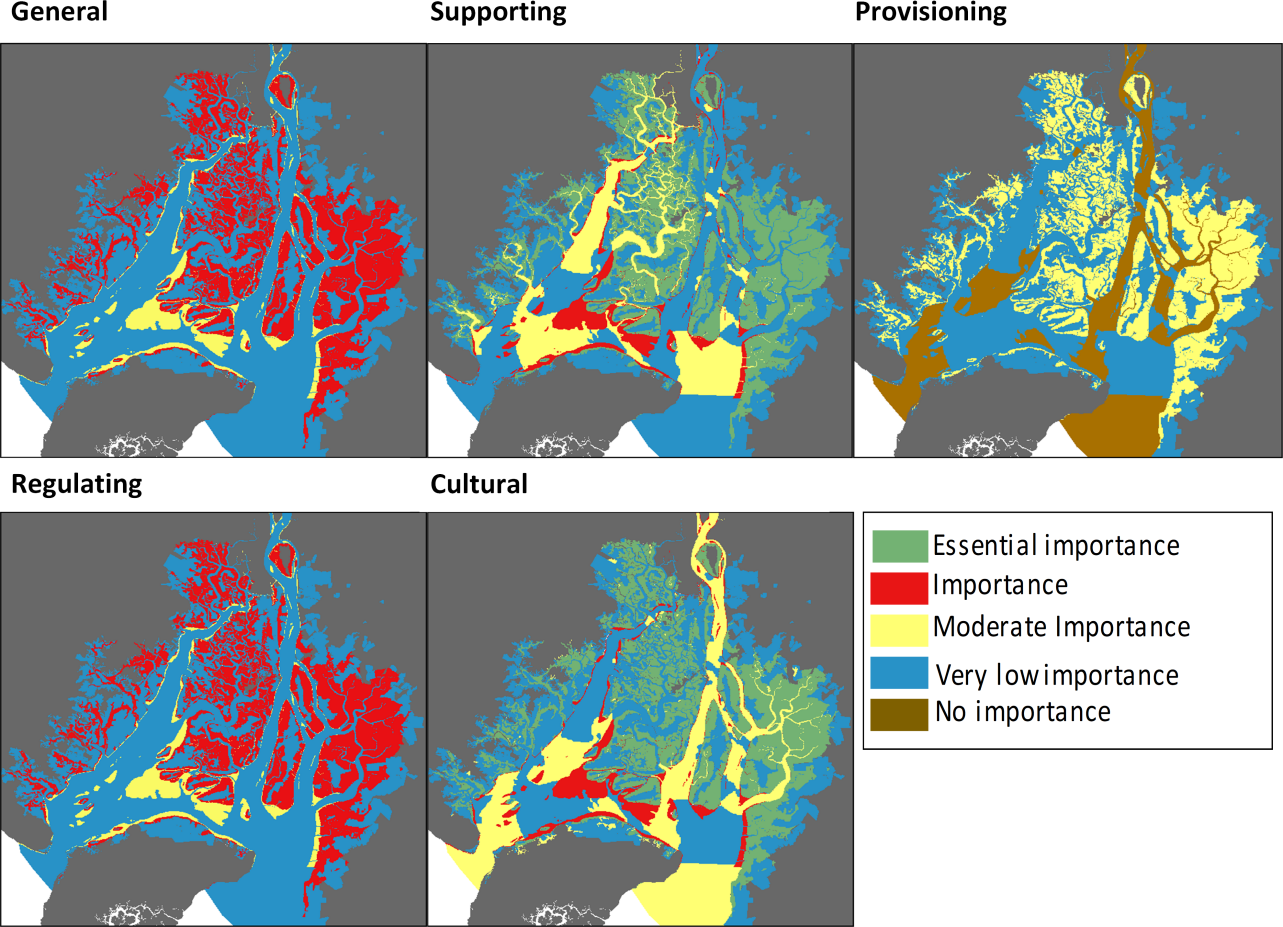
Importance of each habitat in the supply of different types of ecosystem services.

Based on the results shown in Table 3, Fig 6 shows the spatial distribution of the results of the four categories of ES for six of the eight habitats (water column habitats were not represented because they overlap with the sandy and muddy bottoms habitats).

#### b. Determination of the impact of the establishment of protected areas within the study area

Using the current situation analysis together with the results of the first question, the IWG assessed the following question in the third workshop: ¨The habitat… has… capacity to supply the ES… in this zone? ¨. Each IWG member assigned a score from 0 to 5 (0: none, 1: very bad capacity, 2: bad capacity, 3: moderate capacity, 4: good capacity, 5: very good capacity) [57]. Together, with the results of the assessment and considering the common habitats of the zones, a comparison of values obtained in each one of the categories of the ESs from Zones 2, 3, 4 (protected areas) was performed in regards to Zone 1. This analysis was carried out to determine the impact of the establishment of a PA. The final score was determined by applying the average criteria.

**Fig 7:**
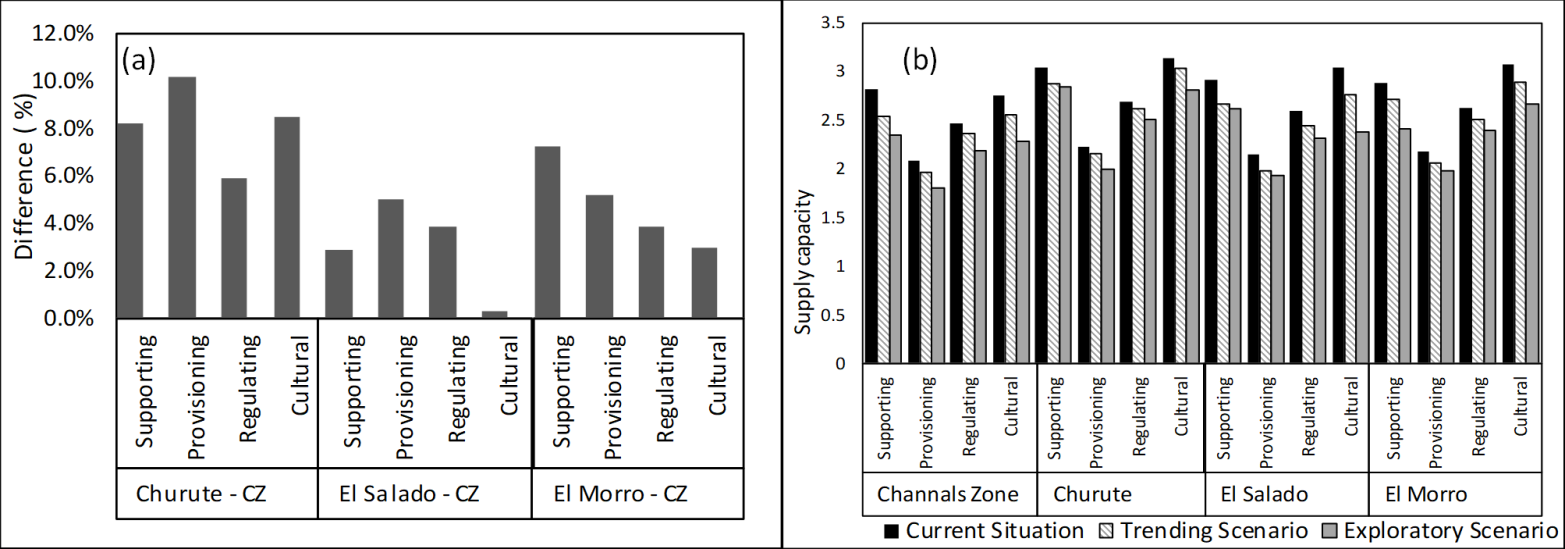
(a) Comparison (%) of the assessment of ESs categories of each of the PAs concerning the Channels Zone (ZC); (b) Assessment of the supply capacity of SE of each zone under different scenarios.

Fig 7A shows the percentage variation of the supply capacity, of each one of the protected areas compared to the Channels Zone (CZ) in the current situation (S5 Table). The PA Churute has the highest percentage of differences regarding Zone 1 than the others PAs. The difference occurs because this PA has received a high degree of awareness and participation from its stakeholders and decision-makers. In addition, it is an area that receives fresh water from various hydrographic basins (the Churute, Taura, Naranjal, Cañar and the low saltwater of the River Guayas) [58]. PA El Morro has a small difference concerning the Channels Zone in regulation and cultural ESs. PA El Salado shows the smallest differences with respect to the Channels Zone, especially in cultural ecosystem services, probably because it is directly affected by the population growth and contamination from the city (being next to the city of Guayaquil), and it has not reached an optimal level of participation and coordination among stakeholders and decision-makers [59,60].

#### c. Assessment of the capacity of habitats in the supply of ecosystem services by scenarios

This assessment was completed for each scenario (trend-based and exploratory) and for each one of the defined zones within the study area, using the same question which was used to evaluate the current situation. During the assessment, it was taken into consideration that the capacity is connected to the quality and quantity in which the service is supplied, which means that an affectation of one or both of these characteristics produces a decrease in the supply capacity of ecosystem services [61].

In Fig 7B, the results of the assessment of the supply capacity of the different ESs categories, in each one of the research zones and scenarios, are represented (S5 Table). It is apparent that in all the study zones, the supply capacity of ESs decreases due to the occurrence of the scenarios. The exploratory scenario has a higher decline incidence in the ecosystem services availability.

**Fig 8:**
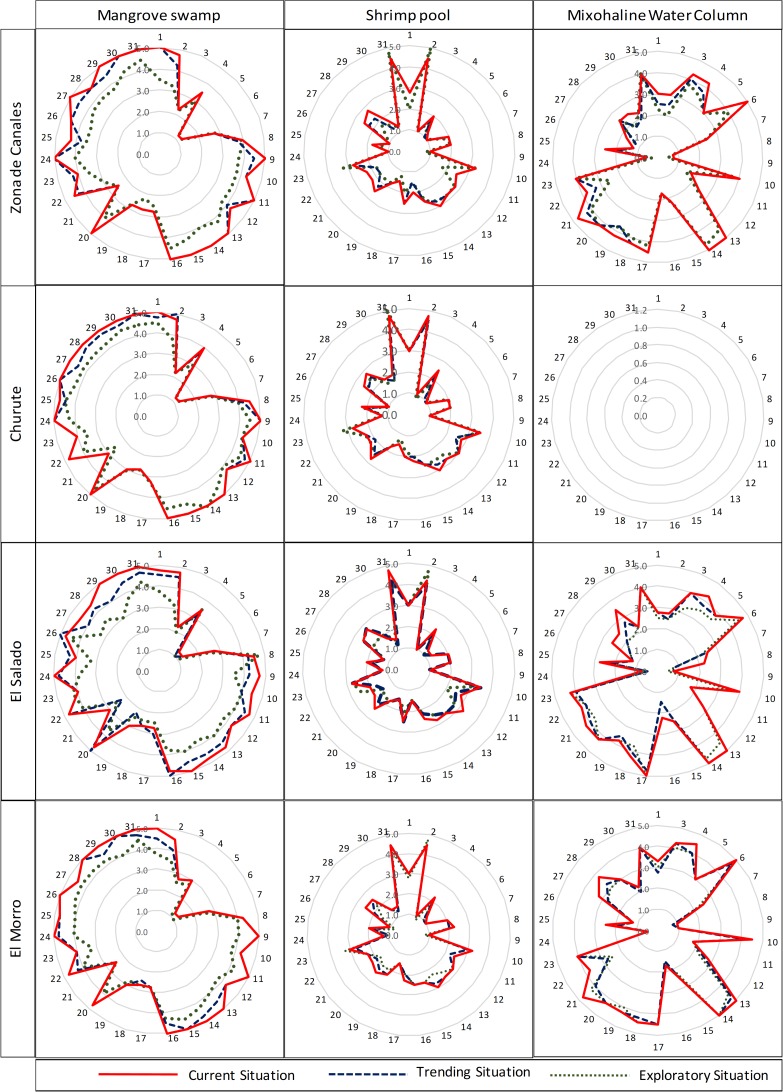
Assessment of the capacity of the ESs by habitat, scenarios, and zones.

In each one of the habitats, an analysis of the supply capacity of each ecosystem service was carried out by observing their behavior in the current situation and the different prospective scenarios, Fig 8. In the trend-based scenario, it is apparent that the contamination and expansion of concrete outwards from the cities have caused a decrease in various services in the different zones of study. The services that were least affected were those related to quality regulation of air and water quantity.

In the exploratory scenario, when the contamination, city size and deforestation of the mangrove forest increased; the expansion of the shrimp habitat produced an increase in both shrimp production and sediment accumulation into the shrimp ponds which later must be removed and used to reinforce the shrimp ponds walls. Therefore, the supply capacity of two ecosystem services (food and the regulation of sedimentation and erosion by the water bodies) increase within the shrimping habitat, thus causing a decrease of other services in the other habitats. As a result, a clear trade-off materialized.

## Discussion

This approach combines the creation of prospective scenarios with the assessment of ecosystem services from the perspective of habitats. This combination allowed us to overcome a drawback from the habitat-based focus in the determination of social demand on the services [25] because when used independently it is centered on ESs, making the determination of the social demand on the services difficult.

Taking into consideration that the ESs are the benefits that human beings obtain from the ecosystem [62] and that there is a close relationship between human uses and natural capital. The social demand was determined by identifying the following: 1) for each human use, which ESs are used, and 2) the most important ESs for each use. The selection of the ESs by human uses allows us to respect the characteristics of each estuary because even though estuaries could share similar natural and ecological features; they could have different socio-economic characteristics determined by human uses.

The scenario building is a powerful tool that allows for visualization and quantification of potential impacts that human uses could have on the ecosystems [63,64], as well as for determination of the trade-offs and synergies that are produced within the system [65]. In this case, the prospective method allowed to systematically create future scenarios for the SA with better spatial and temporal resolution. These scenarios will enable experts who assess the supply capacity of the ESs to have a standard image of the study area, both the current situation and future scenarios.

Since this approach demands that the involved parties complete a lot of matrices, the current situation, the trend-based scenario, and an exploratory scenario were assessed. However, in practice, the prospective allows us to generate a wide range of scenarios that cover the field of likelihood, decreasing uncertainty and establishing a complete diagnosis for the SA [51]. It is for this reason that this method has been incorporated in the strategic planning processes of various Latin American countries [24]. Moreover, its use in the assessment of the ESs will facilitate the inclusion of ecosystems-based-management within public planning and move on to the next stage that is the strategic planning.

In the current situation, the scores of different categories of the ESs within the PAs were higher than those obtained in the Channels Zone. Likewise, it was observed that the assessment results of the PA Churute were superior to the others and the results of the PA El Salado were similar to those of the Channels Zone, which suggests that the effect of the declaration of a PA is not standard throughout the SA. These differences arise because a single statement of a PA is not sufficient enough for environment conservation, as it is necessary for it to be accompanied by a series of actions such as community participation, research, education, management planning, infrastructure development, etc. Another factor to consider is the location of the PA concerning centers of environmental pressure. The PAs are a tool for environmental conservation with economic implications both in the regulation of fishing and the generation of income as a result of tourism [66].

The comparison of the results of the current situation, the exploratory scenario, and the trend-based one shows that the smallest scores were obtained in the exploratory scenario where there is a combined effect of the growth of pollution and a change in the use of land. The comparative analysis of scenarios allows the decision makers to have an image of what strategic actions to implement in order to modify the behavior of the key variables, to reach the desired final state.

In addition to assessing the capacity of habitats in the supply of ecosystem services, the influence of changes in habitats in the key species fate can be analyzed [67]. As an example, in the exploratory scenario, Fig 8 shows an increase of the food within the shrimp habitat and a decrease of the same service in the mangrove habitat. If the analysis is carried out at the species level, it would mean that the reduction of mangrove habitat causes a reduction in *Ucides occidentalis* [68,69]. This crustacean is a key species because it is consumed within the trophic chains of the ecosystem, it is important in the management of litter [27,70], and it is also a traditional fishery influencing the economy of the people who obtain the benefits from this resource in the study area [68,69]. Therefore, a disruption in this key species could produce a societal and ecological negative consequence.

In each workshop, it was important to reach a good representation of the interested sectors in the use of the ecosystem services. In this study, due to the affiliation of the IWG that attended the workshops, the assessment mostly had a public and academic focus. The exchange of information and criteria among participants allowed to reach a better understanding of the problems that exist in the environment and the possible solutions. This approach enables making a general assessment of the system to study and identify hot-spots so that, subsequently, it can assess them with greater detail using other methods such as the process-based one.

## Conclusions

This approach allowed us to determine that the establishment of PA has a positive impact on maintaining the supply of ecosystem services. This impact is not the same in all the PAs because it depends on complementary measures which must be implemented in addition to the PA declaration. The systematic generation of prospective scenarios allowed us to generate alternative futures and with a group of stakeholders assessing the impact on the supply of ecosystem services. These are two tools of the ecosystems-based management that allow the decision-makers to visualize the effects of their decisions and the occurrences of events.

The information generated through this approach will make it easier for decision makers to reach a consensus among interested groups and to move from a passive to an active approach. Making decisions that allow them to achieve the desired effect, which is to maintain the supply of ecosystem services for the people over a long period of time.

## Supporting information

S1 Video Hydrodynamic of the Guayaquil. (RAR)

S1 Dataset Geodatabase of vector and raster files with data of salinity and habitats. (RAR)

S1 Table Human uses/variables of the study area. (PDF)

S2 Table Ecosystem services categories. (PDF)

S3 Table Demand of ecosystem services by human uses. (PDF)

S4 Table Structural Matrix. (XLSX)

S5 Table Assessment of the provision capacity of the ESs by habitat, scenarios, and zones. (PDF)

S1 Fig Causal analysis. (PDF)
