# Supplementary material for: Assessment of ecosystem services of an urbanized tropical estuary with a focus on habitats and scenarios
Source: PLoS One. 2018 Oct 5;13(10):e0203927. doi: 10.1371/journal.pone.0203927 (PMC6173385; doi:10.1371/journal.pone.0203927)
Supplement: S1 Table — (PDF) [file pone.0203927.s003.pdf]

SUPPORTING INFORMATION

S1 Table. HUMAN USES / VARIABLES OF THE STUDY AREA.

SECTORS	HUMAN USES	Num	VARIABLE	DEFINITION	INDICATORS
Conservation	Protected area adjacent to estuarine system	1	Conservation of protected areas adjacent to the estuarine system	Clearly defined, recognized, and managed geographic spaces by legal means or other effective means to achieve the long-term conservation of nature, its ecosystem services, and associated cultural values. Located above the average level of high tides.	Type and extension.
	Protected intertidal/subtidal area	2	Conservation of protected intertidal/subtidal area	Clearly defined, recognized, and managed geographic spaces by legal means or other effective means to achieve the long-term conservation of nature, its ecosystem services, and associated cultural values. Located on the intertidal zone.	Type and extension.
	Protected species	3	Conservation of protected species	Species whose capture, use, collection, and exploitation are prohibited by law. In this case the mangrove swamp.	Extension, and mangrove swamp location
Flood/Coast protection	Setback zones	4	Development of setback zones	Damping zones for deceleration of water bodies. These areas are established through the relocation of infrastructures and the gradual elimination of settlements in the exposed areas. In the case of the PA is considered the area of mangroves and the area that is between protected areas and cities.	Extension of buffer zones.
	Flood bank	5	Development of flood banks.	Structures in the intertidal and subtidal zone designed to reduce the impact of a flood (dam, gabion, wall).	Type of built structures and area of influence.
	Natural Protection	6	Conservation of natural protection	Species or natural geoforms that produce the reduction of energy waves.	Number, location, and area of influence.
Navigation	Capital and construction dredging	7	Development of capital and construction dredging	Capital dredging: When the project of a navigation channel is materialized, the material that exceeds the design depth must be dredged at the first time.<br>Construction dredging: When construction materials are provided (e.g., sand, gravel, shell) or new land is created, for construction.	• Number of capital dredging.<br>• Number of Fillings.
	Maintenance dredging	8	Maintenance of navigation routes	Extraction of material to maintain a navigation route	Amount, periodicity and location of dredging and deposit sites.
	Vessels movement	9	Development of maritime transport	The frequency and size of ships navigating throughout the study system, transporting cargo and passengers. Invasive species are transported in the ballast waters and hulls of ships.	TEUS and number of vessels.
Ports and harbors	Port land claim (intertidal/subtidal)	10	Land claims for port activities	Land claims for port activities during the last 15 years.	Number, location, and extension of claims for port activities.
	Activities related to the port adjacent to the estuarine system.	11	Development of activities related to port operations adjacent to the estuarine system	Activities related to port services that are developed on land.	Extension of area and locations including expansion zones
	Port activity on the intertidal/subtidal area.	12	Development of port activities in the intertidal and subtidal zone.	Activities related to port services that are developed on the intertidal and subtidal zone.	Extension of area and locations.
Infrastructure	Infrastructure on the seabed or in the water column.	13	Development of Infrastructure on the seabed or in the water column.	Construction of infrastructures either on the seabed or in the water column, for different purposes (e.g., pipelines, springs, bridges, etc.).	Number, type, and location of structures
Industry	Water abstraction for industry	14	Access to water abstraction for industry	Water for industrial activity.	Amount of extracted water.
	Aggregate extraction.	15	Development of aggregate extraction	Extraction of aggregates (gravel, sand, etc.) from certain sites, or sediments trapped on land for filling or construction of shrimp walls.	Amount of raw material extracted from the system.
	Industrial discharges.	16	Quality of industrial discharges	The monitoring parameters of the industrial discharges must accomplish with the established levels in UTSEL.	Concentration levels of parameters established in the UTSEL.

	Industrial activity adjacent to the estuarine system	17	Development of Industrial activity adjacent to the estuarine system	Industrial activity established adjacent to the system.	Number of tuna and shrimp packers, shipyards, and vehicle maintenance areas.
Agriculture	Water abstraction for agriculture.	18	Access to water abstraction for agriculture.	Water for irrigation.	Quantity of water for agriculture (ton/year).
	Agricultural run-off	19	Agricultural run-off	The monitoring parameters of the discharges established in the UTSEL, for agricultural activities, accomplish with the established levels.	Concentration levels of parameters established in the UTSEL.
	Soil for agriculture.	20	Occupation of land for agriculture.	Type of crop.	Extension and type of crop in the study area.
Biological extraction	Commercial fishing	21	Extraction of fish and shellfish	Extraction of biological material from the intertidal and subtidal areas for commercial purposes (e.g., fish, seaweed, and shellfish).	Units extracted per year.
Cities and towns	Discharge of wastewater and solid waste	22	Access to wastewater treatment and solid waste management systems.	Access to wastewater treatment systems of domestic origin.<br>Access to domestic solid waste treatment services.	Concentration levels of parameters established in the UTSEL.
	Cities and towns adjacent to the estuarine system	23	Development of cities and towns adjacent to the estuarine system	Cities and towns located adjacent to the system or in the intertidal zone.	Number of dwellings in urban and rural areas within the study area.
	Drinking water	24	Access to drinking water	Water intended for human consumption and domestic use.	Percentage of houses with access to water by a public network, well, delivery car or others.
Aquaculture	Water abstraction for aquaculture.	25	Access to water abstraction for aquaculture.	The water used for shrimp farms must meet the quality criteria for the preservation of flora and fauna in cold or warm fresh waters and marine and estuarine waters established in the UTSEL.	Estimation of the amount of estuarine water taken by the shrimp industry.
	Aquaculture discharge	26	Quality of aquaculture discharge	Discharges of the shrimp farms must accomplish with the established levels, in the UTSEL.	Concentration levels of the parameters established in the UTSEL.
	Soil for aquaculture.	27	Occupation of land for aquaculture	Extension of shrimp farms in the intertidal zone and adjacent to the system.	Extension of the shrimp area.<br>Shrimp production per year.
Tourism, recreation, and nautical sports.	High value landscape	28	Access to High value landscape	Area of outstanding natural beauty (e.g., Parks, landscapes, animal watching).	Estimation of high-value landscape features present within the area as % of outreach/area
	Sport and recreation in the sea, intertidal zone, and subtidal zone	29	Access to sports activities and recreation in the sea, intertidal zone, and subtidal zone	Recreational and sports activities that take place in the intertidal and subtidal zone.	Number of persons performing, per year, activities of bathing, canoeing, sport fishing

UTSEL: Unified Text of Secondary Environmental Legislation

This table was elaborated with the information of the conflict matrix of TIDE project and the coastal marine plan made of SENPLADES.

	TRENDS OF THE ENVIRONMENT	Num	VARIABLE	DEFINITION	INDICATORS
	Climate change	30	Frequency of extreme events.	Identification of hazards due to climate change, frequency, impact, and area of influence.	Number of extreme events of rains and droughts.
	Citizen empowerment and transformation of governments.	31	Organizational capacity development	Development of awareness of what happens in the environment and ability to articulate actions with a specific purpose.	Number of concessions to communes.
