# Supplementary material for: Assessment of ecosystem services of an urbanized tropical estuary with a focus on habitats and scenarios
Source: PLoS One. 2018 Oct 5;13(10):e0203927. doi: 10.1371/journal.pone.0203927 (PMC6173385; doi:10.1371/journal.pone.0203927)
Supplement: S2 Table — (PDF) [file pone.0203927.s004.pdf]

SUPPORTING INFORMATION

S2 Table. ECOSYSTEM SERVICES CATEGORIES

PROVISIONING ESs

No	SERVICES	BENEFITS	SHORT DESCRIPTION
1.1	Food: Plants	Food	Presence and use of edible plants, including agricultural production for direct food consumption
1.2	Food: Animals	Food	presence and use of edible animals, including livestock growth and fodder production
1.3	Water for household use	Drinking water	Provision and use of water for household use meeting the quality standards for drinking water
1.4	Water for industrial use	Improvement of Industrial production, allows industrial development and improvement of production.	Provision and use of water for e.g. cooling water, rinsing water, water for chemical reactions
1.5	Water for aquaculture use	Shrimp farming	Use of salt water with certain quality conditions for shrimp farming
1.6	Water for agricultural use	Improved agricultural production	Provision and use of water for e.g. irrigation water, freezing prevention for fruit trees, drinking water for cattle
1.7	Water for energy use	Renewable energy production	provision and use of water for tidal or dam water turbines
1.8	Water for navigation	Shipping	Presence and use of water for shipping purposes
1.9	Raw materials: Renewable soil materials: sand	Building material	Provision and use of sand from dynamic environments which are renewed within a few generations (100 y)
1.10	Raw materials: Renewable soil materials: clay	Building material	Provision and use of sand from dynamic environments which are renewed within a few generations (100 y)
1.11	Raw materials: Platform	Building platform for housing, roads infrastructure, shrimp pools	presence and use of stable and safe environments for building of infrastructure: housing, roads, shrimp pools
1.12	Raw materials: Plants	Building material, fiber, fuel	Presence and use of forests, energy, and fiber crops
1.13	Raw materials: Animals	Building material, fiber, fuel	presence and use of animals for fur, leather, gelatin,
1.14	Genetic resources	Various improved provisioning services.	Presence and use of typical varieties and cultivars of species, adapted to a specific environment
1.15	Medicinal resources	Human health	Presence and use of plants/organisms used in herbal medicine, medicinal tea,...
1.16	Ornamental resources	Wellbeing	Presence and use of organisms for decorative purposes
1.17	Fossil energy sources	Energy for the movement of machinery and vehicles	Extraction and use of oil or natural gas for human activities.

REGULATING ESs

No	SERVICES	BENEFITS	SHORT DESCRIPTION
2.1	AQR: Removing harmful particles	Human health	Adsorption of fine dust and pollutants on leaf surfaces of forests,…
2.2	AQR: Air-water exchange	Human health	influence of evaporation and evapotranspiration, condensation on air quality
2.3	AQR: Biogeochemical reactions due to activity of organisms	Human health	respiration and photosynthesis, exudation of chemicals by degradation reactions
2.4	CR: Carbon sequestration and burial	Human health, avoided costs caused by extreme events or disturbance, ensured provisioning services	buffering carbon stock in living vegetation, burial of organic matter in soils
2.5	CR: Water thermodynamic regulation	Human health, avoided costs caused by extreme events or disturbance, ensured provisioning services	cooling effect of vegetation, uptake of solar energy for photosynthesis and evapotranspiration,
2.6	CR: Heat exchange regulation	human health, avoided costs caused by extreme events or disturbance, ensured provisioning services	Effect of direct reflection, storage, transport, radiation of solar heat by various soil and water bodies
2.7	REE: Flood water storage	human health, avoided costs caused by extreme events or disturbance, ensured provisioning services	storage of storm or extreme spring tides in natural or flood control habitats
2.8	REE: Peak discharge buffering	Human health, avoided costs caused by extreme events or disturbance, ensured provisioning services	storage of peak discharge floods in natural or flood control habitats
2.9	REE: Water current reduction	Human health, avoided costs caused by extreme events or disturbance, ensured provisioning services	Reduction of water current by physical features or vegetation
2.10	REE: Wave reduction	Human health, avoided costs caused by extreme events or disturbance, ensured provisioning services	Reduction of wave height by physical features (diffraction at the mouth of the estuary) or vegetation
2.11	REE: Sound buffering	Human health	Reduction of noise disturbance by presence of natural buffers
2.12	WQR: Drainage of river water	Ensured platform, food, water other provisioning services	Drainage of the catchment by the river
2.13	WQR: Prevention of saline intrusion	Various ensured provisioning services	Countering of saline tidal wave by fresh water discharge
2.14	WQR: Dissipation of tidal and river energy	Various ensured provisioning services, avoided maintenance costs	Buffering of average flood and discharge variations in the river bed
2.15	WQR: Landscape maintenance	Various ensured services	Formation and maintenance of typical landscapes and hydrology
2.16	WQR: Transportation	Shipping	Discharge and tidal input for shipping, including water use for canals and docks
2.17	WQlR: Transport of pollutants and excess nutrients	Improved water quality, various ensured services	Transport of pollutants from source, dilution
2.18	WQlR: reduction of excess loads coming from the catchment	Improved water quality, various ensured services	Binding of N, P in sediments and pelagic food web
2.19	Erosion and sedimentation regulation by water bodies	Avoided damage or maintenance costs, various ensured provisioning services	Sediment trapping and gully erosion by variable water currents and topography
2.20	Erosion and sedimentation regulation by biological mediation	Avoided damage or maintenance costs, various ensured provisioning services	Sediment trapping and erosion prevention by vegetation, effects of bioturbation
2.21	Biological regulation of soil processes and soil formation.	Various ensured provisioning services	Soil microbial activities important for agriculture or water quality regulation processes, bioturbation
2.22	Prevention of establishment of harmful invasive species	Various ensured provisioning services	Presence of resilient natural populations able to withstand invasion

2.23	Reduced spread of diseases	Various ensured provisioning services, human health	Presence of resilient and equilibrated natural populations avoiding excessive population growth of disease-carrying vector species, importance for human health or agriculture.
2.24	Pollination	Various ensured provisioning services	Presence of pollinators and importance for agricultural production and mangroves.
2.25	Pest control	Various ensured provisioning services	Presence of predators for problematic pest species impacting agricultural production

SUPPORTING ESs

3.1	Biodiversity	Assurance of all services	Total amount of abiotic and biotic diversity at all levels (gene-landscape), regardless of rarity or vulnerability

CULTURAL ESs

4.1	Aesthetic information	Wellbeing	Appreciation of beauty of organisms, landscapes,
4.2	Opportunities for recreation & tourism	Wellbeing	Opportunities and exploitation for recreation & tourism
4.3	Inspiration for culture, art and design	Wellbeing	Appreciation of organisms, landscapes, as inspiration for culture, art, and design
4.4	Spiritual experience	Wellbeing	Appreciation of organisms, landscapes, on a spiritual level
4.5	Information for cognitive development	Wellbeing	Use of organisms, landscapes for (self-) educational purposes

AQR: Air quality regulation; CR: Climate regulation; REE: Regulation extreme events or disturbance; WQR: Water quantity regulation; WQIR: Water quality regulation

This table was taken from the TIDE project. Some services were modified and two ESs were added to the list. http://www.tide-project.eu/downloads/ES_PDF_KW.pdf
