# Supplementary material for: Assessment of ecosystem services of an urbanized tropical estuary with a focus on habitats and scenarios
Source: PLoS One. 2018 Oct 5;13(10):e0203927. doi: 10.1371/journal.pone.0203927 (PMC6173385; doi:10.1371/journal.pone.0203927)
Supplement: S3 Table — (PDF) [file pone.0203927.s005.pdf]

SUPPORTING INFORMATION

S3 Table. DEMAND OF ECOSYSTEM SERVICES BY HUMAN USES

		3.1	1.2	1.4	1.5	1.7	1.8	1.9	1.11	2.1	2.2	2.4	2.6	2.7	2.8	2.9	2.10	2.12	2.13	2.14	2.15	2.17	2.18	2.19	2.20.	2.22	2.24	4.1	4.2	4.3	4.4	4.5
		A	P	P	P	P	P	P	P	R	R	R	R	R	R	R	R	R	R	R	R	R	R	R	R	R	R	C	C	C	C	C
Num		Biodiversity	Food: Animals	Water for industrial uses	Water for aquaculture	Water for energy use	Water for navigation	Raw materials: Renewable soil materials: sand	Raw materials: Platform	AQR: Removing harmful particles	AQR: Air-water exchange	CR: Carbon sequestration and burial	CR: Heat exchange regulation	REE: Flood water storage	REE: Peak discharge buffering	REE: Water current reduction	REE: Wave reduction	WQR: Drainage of river water	WQR: Prevention of saline intrusion.	WQR: Dissipation of tidal and river energy energy	WQR: Landscape maintenance	WQIR: Transport of pollutants and excess nutrients	WQIR: Reduction of excess loads coming from the catchment	Erosion and sedimentation regulation by water bodies	Erosion and sedimentation regulation by biological mediation	Prevention of establishment of harmful invasive species	Pollination	Aesthetic information	Opportunities for recreation & tourism	Inspiration for culture, art, and design	Spiritual experience	Information for cognitive development
1	Protected area adjacent to estuarine system	3																				3					3	3	3	3	3	3
2	Protected intertidal/subtidal area	3															3		3			3		3			3	3	3	3	3	3
3	Protected species	3															3		3			3		3			3	3	3	3	3	3
4	Setback zones	3									3				3	3						3			3			3	3	3	3	3
5	Flood bank													3	3	3	3	3		3				3								
6	Natural Protection																3															3
7	Capital and construction dredging	3					3	3										3						3								3
8	Maintenance Dredging	3					3	3										3						3	3							3
9	Vessels movement	3					3													3	3			3					3	3		3
10	Port land claim (intertidal/subtidal)						3		3																							3

11	Activities related to the port adjacent to the estuarine system.						3		3																							
12	Port activity on the intertidal / subtidal area.						3									3	3			3												
13	Infrastructure on the seabed or in the water column.						3		3							3	3															3
14	Water abstraction for industry			3		3																										
15	Aggregate extraction.							3												3				3								
16	Industrial discharges.			3		3							3									3	3									3
17	Industrial activity adjacent to the estuarine system			3		3						3																				
18	Water abstraction for agriculture.	3																														
19	Agricultural run-off																					3	3									
20	Soil for agriculture.	3							3																							
21	Commercial fishing	3	3												3										3	3				3		3
22	Discharge of waste water and solid waste	3																				3	3									
23	Cities and towns adjacent to the estuarine system								3	3	3		3															3			3	
24	Drinking water	3																	3													
25	Water abstraction for aquaculture.				3														3	3		3	3									3
26	Aquaculture discharge																		3			3	3									3
27	Soil for aquaculture.				3			3	3						3				3	3				3	3	3						3
28	High value landscape	3	3																		3							3	3	3	3	3
29	Sport and recreation in the sea, intertidal zone, and subtidal zone						3															3	3					3	3	3	3	3
	**Total**	**39**	**6**	**9**	**6**	**9**	**24**	**12**	**18**	**3**	**6**	**3**	**6**	**3**	**12**	**12**	**18**	**9**	**18**	**18**	**6**	**30**	**18**	**24**	**12**	**6**	**9**	**21**	**21**		**21**	**51**

This table shows the services that reach the maximum score.
