# Supplementary material for: Assessment of ecosystem services of an urbanized tropical estuary with a focus on habitats and scenarios
Source: PLoS One. 2018 Oct 5;13(10):e0203927. doi: 10.1371/journal.pone.0203927 (PMC6173385; doi:10.1371/journal.pone.0203927)
Supplement: S5 Table — (PDF) [file pone.0203927.s007.pdf]

## SUPPORTING INFORMATION

### S5 Table. Current Situation-Zone 1

| | 1 | 2 | 3 | 4 | 5 | 6 | 7 | 8 | 9 | 10 | 11 | 12 | 13 | 14 | 15 | 16 | 17 | 18 | 19 | 20 | 21 | 22 | 23 | 24 | 25 | 26 | 27 | 28 | 29 | 30 | 31 |
|---|---|---|---|---|---|---|---|---|---|---|---|---|---|---|---|---|---|---|---|---|---|---|---|---|---|---|---|---|---|---|---|
| | 3.1 | 1.2 | 1.4 | 1.5 | 1.7 | 1.8 | 1.9 | 1.11 | 2.1 | 2.2 | 2.4 | 2.6 | 2.7 | 2.8 | 2.9 | 2.10. | 2.12 | 2.13 | 2.14 | 2.15 | 2.17 | 2.18 | 2.19 | 2.20. | 2.22 | 2.24 | 4.1 | 4.2 | 4.3 | 4.4 | 4.5 |
| | A | P | P | P | P | P | P | P | R | R | R | R | R | R | R | R | R | R | R | R | R | R | R | R | R | R | C | C | C | C | C |
| | Biodiversity | Food: Animals | Water for industrial uses | Water for aquaculture | Water for energy use | Water for navigation | Raw materials: Renewable soil materials: sand materials: sand | Raw materials: Platform | AQR: Removing harmful particles | AQR: Air-water exchange | CR: Carbon sequestration and burial | CR: Heat exchange regulation | REE: Flood water storage | REE: Peak discharge buffering | REE: Water current reduction | REE: Wave reduction | WQR: Drainage of river water | WQR: Prevention of saline intrusion. | WQR: Dissipation of tidal and river energy | WQR: Landscape maintenance | WQIR: Transport of pollutants and excess nutrients | WQIR: Reduction of excess loads coming from the catchment | Erosion and sedimentation regulation by water bodies | Erosion and sedimentation regulation by biological mediation | Prevention of establishment of harmful invasive species | Pollination | Aesthetic information | Opportunities for recreation & tourism | Inspiration for culture, art, and design | Spiritual experience | Information for cognitive development |
| Salt flats | 2.8 | 2.0 | 0.5 | 0.5 | 0.8 | 0.5 | 1.5 | 4.3 | 2.0 | 0.8 | 1.0 | 1.5 | 1.8 | 1.5 | 1.5 | 1.3 | 1.8 | 1.0 | 1.0 | 2.3 | 1.0 | 1.3 | 1.5 | 1.3 | 1.8 | 1.5 | 1.8 | 2.3 | 1.5 | 1.5 | 4.3 |
| Mangrove swamp | 5.0 | 4.8 | 2.3 | 3.5 | 1.3 | 1.3 | 2.8 | 4.0 | 5.0 | 4.3 | 5.0 | 4.3 | 5.0 | 5.0 | 5.0 | 5.0 | 2.8 | 2.8 | 2.8 | 5.0 | 2.5 | 4.5 | 4.0 | 5.0 | 4.3 | 4.5 | 5.0 | 4.5 | 5.0 | 5.0 | 5.0 |
| Shrimp pool | 2.8 | 4.5 | 1.0 | 2.0 | 1.3 | 1.0 | 2.0 | 2.0 | 1.0 | 3.3 | 2.5 | 2.8 | 2.8 | 3.0 | 2.5 | 2.0 | 2.5 | 1.5 | 1.8 | 2.5 | 2.3 | 2.3 | 2.8 | 1.0 | 1.8 | 1.0 | 2.5 | 2.8 | 1.8 | 1.3 | 4.5 |
| Sand and mudflats | 2.0 | 2.3 | 0.5 | 0.8 | 0.5 | 1.8 | 2.5 | 2.8 | 0.5 | 1.0 | 1.8 | 1.8 | 3.5 | 3.3 | 3.5 | 2.8 | 3.5 | 1.3 | 1.8 | 3.8 | 1.3 | 2.0 | 3.5 | 2.5 | 2.0 | 1.0 | 3.0 | 3.0 | 2.5 | 2.5 | 4.3 |
| Sandy bottoms subtidal | 1.8 | 2.3 | 0.5 | 1.0 | 0.5 | 0.5 | 3.8 | 1.5 | 0.5 | 0.8 | 1.5 | 1.5 | 1.0 | 1.3 | 1.5 | 1.5 | 3.8 | 1.5 | 1.5 | 1.8 | 1.3 | 2.0 | 0.5 | 1.5 | 2.8 | 0.8 | 2.0 | 1.5 | 1.8 | 1.8 | 4.3 |
| Muddy bottoms subtidal | 1.5 | 2.3 | 0.5 | 0.5 | 0.5 | 0.5 | 2.0 | 2.0 | 0.8 | 1.0 | 2.3 | 1.8 | 1.3 | 1.5 | 1.8 | 1.5 | 3.8 | 1.5 | 2.0 | 2.0 | 1.3 | 2.3 | 2.0 | 1.5 | 2.5 | 0.8 | 1.8 | 1.8 | 1.3 | 1.3 | 3.8 |
| Oligo-Mesohaline water column | 3.8 | 3.5 | 4.3 | 4.0 | 2.3 | 4.0 | 2.5 | 0.8 | 0.8 | 4.5 | 1.8 | 3.0 | 5.0 | 5.0 | 2.5 | 1.8 | 5.0 | 4.5 | 4.3 | 4.5 | 4.8 | 3.8 | 4.5 | 0.5 | 2.3 | 1.3 | 1.8 | 2.8 | 2.5 | 1.3 | 4.3 |
| Polyhaline water column | 3.0 | 3.0 | 4.3 | 4.3 | 3.0 | 5.0 | 2.5 | 0.8 | 0.8 | 4.0 | 1.8 | 2.5 | 5.0 | 5.0 | 2.3 | 1.8 | 4.5 | 4.3 | 4.3 | 4.3 | 4.8 | 3.8 | 4.0 | 0.5 | 2.5 | 1.3 | 1.8 | 2.5 | 2.5 | 2.3 | 4.0 |
| | 2.8 | 3.1 | 1.7 | 2.1 | 1.3 | 1.8 | 2.4 | 2.3 | 1.4 | 2.4 | 2.2 | 2.4 | 3.2 | 3.2 | 2.6 | 2.2 | 3.4 | 2.3 | 2.4 | 3.3 | 2.4 | 2.7 | 2.8 | 1.7 | 2.5 | 1.5 | 2.4 | 2.6 | 2.3 | 2.1 | 4.3 |
| | 2.81 | 2.08 | | | | | | | 2.47 | | | | | | | | | | | | | | | | | | 2.76 | | | | |

## S5 Table. Current Situation- Churute PA

| | 1 | 2 | 3 | 4 | 5 | 6 | 7 | 8 | 9 | 10 | 11 | 12 | 13 | 14 | 15 | 16 | 17 | 18 | 19 | 20 | 21 | 22 | 23 | 24 | 25 | 26 | 27 | 28 | 29 | 30 | 31 |
|---|---|---|---|---|---|---|---|---|---|---|---|---|---|---|---|---|---|---|---|---|---|---|---|---|---|---|---|---|---|---|---|
| | 3.1 | 1.2 | 1.4 | 1.5 | 1.7 | 1.8 | 1.9 | 1.11. | 2.1 | 2.2 | 2.4 | 2.6 | 2.7 | 2.8 | 2.9 | 2.10. | 2.12 | 2.13 | 2.14 | 2.15 | 2.17 | 2.18 | 2.19 | 2.20. | 2.22 | 2.24 | 4.1 | 4.2 | 4.3 | 4.4 | 4.5 |
| | A | P | P | P | P | P | P | P | R | R | R | R | R | R | R | R | R | R | R | R | R | R | R | R | R | R | C | C | C | C | C |
| | Biodiversity | Food: Animals | Water for industrial uses | Water for aquaculture | Water for energy use | Water for navigation | Raw materials: Renewable soil materials: sand materials: sand | Raw materials: Platform | AQR: Removing harmful particles | AQR: Air-water exchange | CR: Carbon sequestration and burial | CR: Heat exchange regulation | REE: Flood water storage | REE: Peak discharge buffering | REE: Water current reduction | REE: Wave reduction | WQR: Drainage of river water | WQR: Prevention of saline intrusion. | WQR: Dissipation of tidal and river energy | WQR: Landscape maintenance | WQIR: Transport of pollutants and excess nutrients | WQIR: Reduction of excess loads coming from the catchment | Erosion and sedimentation regulation by water bodies | Erosion and sedimentation regulation by biological mediation | Prevention of establishment of harmful invasive species | Pollination | Aesthetic information | Opportunities for recreation & tourism | Inspiration for culture, art, and design | Spiritual experience | Information for cognitive development |
| Salt flats | | | | | | | | | | | | | | | | | | | | | | | | | | | | | | | |
| Mangrove swamp | 5.0 | 4.8 | 2.3 | 4.0 | 1.3 | 1.3 | 2.8 | 4.5 | 5.0 | 4.3 | 5.0 | 4.3 | 5.0 | 5.0 | 5.0 | 5.0 | 3.3 | 2.8 | 3.0 | 5.0 | 3.0 | 4.8 | 4.0 | 5.0 | 4.8 | 5.0 | 5.0 | 5.0 | 5.0 | 5.0 | 5.0 |
| Shrimp pool | 3.0 | 4.8 | 1.0 | 2.5 | 1.3 | 1.0 | 2.0 | 2.0 | 1.0 | 3.5 | 2.8 | 3.0 | 2.8 | 3.0 | 2.5 | 2.3 | 2.0 | 1.5 | 1.8 | 2.8 | 2.3 | 2.3 | 2.8 | 1.3 | 2.3 | 1.0 | 2.5 | 2.8 | 2.0 | 2.0 | 4.8 |
| Sand and mudflats | 2.8 | 3.8 | 0.8 | 1.0 | 0.8 | 2.3 | 3.0 | 3.0 | 1.0 | 1.5 | 2.0 | 2.0 | 2.5 | 2.3 | 3.5 | 3.0 | 3.5 | 1.3 | 2.0 | 2.8 | 2.0 | 2.0 | 3.3 | 2.5 | 3.0 | 1.8 | 3.3 | 3.3 | 2.8 | 3.0 | 4.3 |
| Sandy bottoms subtidal | 2.0 | 2.8 | 0.8 | 1.0 | 0.5 | 0.8 | 4.3 | 1.8 | 0.8 | 0.8 | 1.8 | 1.8 | 1.0 | 1.5 | 1.8 | 1.5 | 3.8 | 1.5 | 1.8 | 2.3 | 1.3 | 2.0 | 0.8 | 1.8 | 3.5 | 1.0 | 2.0 | 2.3 | 2.0 | 1.8 | 4.5 |
| Muddy bottoms subtidal | 1.8 | 2.3 | 0.8 | 0.8 | 0.5 | 0.8 | 2.0 | 2.0 | 1.0 | 1.0 | 2.8 | 2.0 | 1.5 | 1.5 | 2.0 | 1.5 | 3.8 | 1.5 | 2.0 | 2.5 | 1.5 | 2.5 | 2.3 | 1.8 | 3.3 | 1.0 | 2.0 | 2.0 | 1.3 | 1.5 | 4.0 |
| Oligo-Mesohaline water column | 3.8 | 3.5 | 4.3 | 5.0 | 3.0 | 4.0 | 2.5 | 0.8 | 1.0 | 4.5 | 1.8 | 3.3 | 5.0 | 5.0 | 2.8 | 2.0 | 5.0 | 4.8 | 4.3 | 4.8 | 5.0 | 4.0 | 4.8 | 1.0 | 3.3 | 1.8 | 2.0 | 4.0 | 2.5 | 2.5 | 4.3 |
| Polyhaline water column | | | | | | | | | | | | | | | | | | | | | | | | | | | | | | | |
| | 3.0 | 3.6 | 1.6 | 2.4 | 1.2 | 1.7 | 2.8 | 2.3 | 1.6 | 2.6 | 2.7 | 2.7 | 3.0 | 3.0 | 2.9 | 2.5 | 3.5 | 2.2 | 2.5 | 3.3 | 2.5 | 2.9 | 3.0 | 2.2 | 3.3 | 1.9 | 2.8 | 3.2 | 2.6 | 2.6 | 4.5 |
| | 3.04 | 2.23 | | | | | | | 2.69 | | | | | | | | | | | | | | | | | | 3.13 | | | | |

## S5 Table. Current Situation - El Salado PA

| | 1 | 2 | 3 | 4 | 5 | 6 | 7 | 8 | 9 | 10 | 11 | 12 | 13 | 14 | 15 | 16 | 17 | 18 | 19 | 20 | 21 | 22 | 23 | 24 | 25 | 26 | 27 | 28 | 29 | 30 | 31 |
|---|---|---|---|---|---|---|---|---|---|---|---|---|---|---|---|---|---|---|---|---|---|---|---|---|---|---|---|---|---|---|---|
| | 3.1 | 1.2 | 1.4 | 1.5 | 1.7 | 1.8 | 1.9 | 1.11. | 2.1 | 2.2 | 2.4 | 2.6 | 2.7 | 2.8 | 2.9 | 2.10. | 2.12 | 2.13 | 2.14 | 2.15 | 2.17 | 2.18 | 2.19 | 2.20. | 2.22 | 2.24 | 4.1 | 4.2 | 4.3 | 4.4 | 4.5 |
| | A | P | P | P | P | P | P | P | R | R | R | R | R | R | R | R | R | R | R | R | R | R | R | R | R | R | C | C | C | C | C |
| | Biodiversity | Food: Animals | Water for industrial uses | Water for aquaculture | Water for energy use | Water for navigation | Raw materials: Renewable soil materials: sand materials: sand | Raw materials: Platform | AQR: Removing harmful particles | AQR: Air-water exchange | CR: Carbon sequestration and burial | CR: Heat exchange regulation | REE: Flood water storage | REE: Peak discharge buffering | REE: Water current reduction | REE: Wave reduction | WQR: Drainage of river water | WQR: Prevention of saline intrusion. | WQR: Dissipation of tidal and river energy | WQR: Landscape maintenance | WQIR: Transport of pollutants and excess nutrients | WQIR: Reduction of excess loads coming from the catchment | Erosion and sedimentation regulation by water bodies | Erosion and sedimentation regulation by biological mediation | Prevention of establishment of harmful invasive species | Pollination | Aesthetic information | Opportunities for recreation & tourism | Inspiration for culture, art, and design | Spiritual experience | Information for cognitive development |
| Salt flats | 2.5 | 1.5 | 0.5 | 0.5 | 0.8 | 0.5 | 1.5 | 4.8 | 2.0 | 0.8 | 1.5 | 2.0 | 2.0 | 1.8 | 1.8 | 1.5 | 1.5 | 1.3 | 1.3 | 3.0 | 1.3 | 1.5 | 1.8 | 1.3 | 1.8 | 1.8 | 2.3 | 2.8 | 1.8 | 1.5 | 4.3 |
| Mangrove swamp | 4.8 | 4.8 | 2.3 | 3.5 | 1.3 | 1.3 | 2.8 | 4.5 | 4.8 | 4.5 | 4.8 | 4.3 | 4.8 | 4.8 | 5.0 | 4.8 | 2.8 | 2.8 | 3.0 | 4.8 | 3.0 | 4.8 | 4.0 | 5.0 | 4.0 | 4.8 | 4.5 | 4.5 | 5.0 | 5.0 | 5.0 |
| Shrimp pool | 3.0 | 4.3 | 1.0 | 2.3 | 1.3 | 1.3 | 2.0 | 2.0 | 1.0 | 3.5 | 2.8 | 3.3 | 2.8 | 2.8 | 2.5 | 1.5 | 2.5 | 1.5 | 1.8 | 2.5 | 2.0 | 2.0 | 2.8 | 1.3 | 2.0 | 1.3 | 2.5 | 2.8 | 1.8 | 1.5 | 4.8 |
| Sand and mudflats | 2.3 | 2.5 | 0.8 | 1.0 | 0.8 | 2.0 | 2.5 | 2.8 | 0.8 | 1.3 | 1.8 | 2.0 | 2.8 | 3.3 | 3.5 | 2.8 | 3.5 | 1.5 | 2.3 | 3.5 | 1.8 | 1.8 | 3.3 | 3.0 | 2.3 | 1.0 | 3.3 | 3.0 | 2.8 | 2.8 | 4.3 |
| Sandy bottoms subtidal | | | | | | | | | | | | | | | | | | | | | | | | | | | | | | | |
| Muddy bottoms subtidal | 2.3 | 2.3 | 0.8 | 0.8 | 0.8 | 0.8 | 2.0 | 2.0 | 0.8 | 1.0 | 2.3 | 2.0 | 1.5 | 1.8 | 2.0 | 1.5 | 3.8 | 1.5 | 2.0 | 2.5 | 1.8 | 2.8 | 2.5 | 1.8 | 2.5 | 1.0 | 2.0 | 2.0 | 1.3 | 1.5 | 3.8 |
| Oligo-Mesohaline water column | | | | | | | | | | | | | | | | | | | | | | | | | | | | | | | |
| Polyhaline water column | 2.8 | 2.8 | 4.0 | 4.3 | 3.8 | 4.8 | 2.5 | 2.3 | 0.8 | 4.0 | 1.8 | 2.8 | 5.0 | 5.0 | 2.5 | 2.3 | 5.0 | 4.3 | 3.8 | 4.3 | 4.3 | 4.0 | 4.3 | 0.8 | 2.8 | 1.3 | 2.5 | 2.5 | 3.5 | 2.3 | 4.0 |
| | 2.9 | 3.0 | 1.5 | 2.0 | 1.4 | 1.8 | 2.2 | 3.0 | 1.7 | 2.5 | 2.5 | 2.7 | 3.1 | 3.2 | 2.9 | 2.4 | 3.2 | 2.1 | 2.3 | 3.4 | 2.3 | 2.8 | 3.1 | 2.2 | 2.5 | 1.8 | 2.8 | 2.9 | 2.7 | 2.4 | 4.3 |
| | 2.92 | 2.14 | | | | | | | 2.59 | | | | | | | | | | | | | | | | | | 3.03 | | | | |

## S5 Table. Current Situation - El Morro PA

| | 1 | 2 | 3 | 4 | 5 | 6 | 7 | 8 | 9 | 10 | 11 | 12 | 13 | 14 | 15 | 16 | 17 | 18 | 19 | 20 | 21 | 22 | 23 | 24 | 25 | 26 | 27 | 28 | 29 | 30 | 31 |
|---|---|---|---|---|---|---|---|---|---|---|---|---|---|---|---|---|---|---|---|---|---|---|---|---|---|---|---|---|---|---|---|
| | 3.1 | 1.2 | 1.4 | 1.5 | 1.7 | 1.8 | 1.9 | 1.11. | 2.1 | 2.2 | 2.4 | 2.6 | 2.7 | 2.8 | 2.9 | 2.10. | 2.12 | 2.13 | 2.14 | 2.15 | 2.17 | 2.18 | 2.19 | 2.20. | 2.22 | 2.24 | 4.1 | 4.2 | 4.3 | 4.4 | 4.5 |
| | A | P | P | P | P | P | P | P | R | R | R | R | R | R | R | R | R | R | R | R | R | R | R | R | R | R | C | C | C | C | C |
| | Biodiversity | Food: Animals | Water for industrial uses | Water for aquaculture | Water for energy use | Water for navigation | Raw materials: Renewable soil materials: sand materials: sand | Raw materials: Platform | AQR: Removing harmful particles | AQR: Air-water exchange | CR: Carbon sequestration and burial | CR: Heat exchange regulation | REE: Flood water storage | REE: Peak discharge buffering | REE: Water current reduction | REE: Wave reduction | WQR: Drainage of river water | WQR: Prevention of saline intrusion. | WQR: Dissipation of tidal and river energy | WQR: Landscape maintenance | WQIR: Transport of pollutants and excess nutrients | WQIR: Reduction of excess loads coming from the catchment | Erosion and sedimentation regulation by water bodies | Erosion and sedimentation regulation by biological mediation | Prevention of establishment of harmful invasive species | Pollination | Aesthetic information | Opportunities for recreation & tourism | Inspiration for culture, art, and design | Spiritual experience | Information for cognitive development |
| Salt flats | | | | | | | | | | | | | | | | | | | | | | | | | | | | | | | |
| Mangrove swamp | 5.0 | 4.5 | 2.8 | 3.0 | 1.3 | 1.3 | 2.8 | 4.3 | 5.0 | 4.3 | 5.0 | 4.3 | 5.0 | 5.0 | 5.0 | 5.0 | 2.8 | 2.8 | 3.0 | 4.8 | 2.5 | 4.8 | 4.0 | 5.0 | 4.8 | 5.0 | 4.5 | 5.0 | 5.0 | 5.0 | 5.0 |
| Shrimp pool | 3.0 | 4.5 | 1.0 | 2.3 | 1.3 | 1.0 | 2.0 | 2.3 | 1.0 | 3.3 | 2.5 | 2.8 | 2.8 | 3.0 | 2.5 | 2.5 | 2.3 | 1.5 | 2.3 | 2.5 | 2.3 | 2.3 | 3.0 | 1.3 | 2.0 | 1.0 | 2.5 | 2.5 | 1.5 | 1.5 | 4.5 |
| Sand and mudflats | 2.5 | 2.5 | 0.8 | 1.0 | 0.8 | 2.0 | 2.8 | 2.8 | 1.0 | 1.5 | 1.8 | 1.8 | 3.5 | 3.3 | 3.5 | 3.8 | 3.5 | 1.5 | 2.3 | 3.8 | 1.5 | 2.3 | 3.5 | 2.8 | 2.3 | 1.3 | 3.5 | 3.5 | 2.8 | 2.8 | 4.5 |
| Sandy bottoms subtidal | 1.8 | 2.8 | 0.8 | 1.3 | 0.8 | 0.5 | 4.3 | 1.5 | 0.8 | 1.0 | 1.8 | 1.8 | 1.3 | 1.3 | 1.8 | 1.5 | 3.8 | 1.5 | 1.8 | 2.0 | 1.5 | 2.0 | 0.8 | 1.5 | 3.0 | 1.0 | 2.0 | 1.8 | 1.8 | 1.8 | 4.3 |
| Muddy bottoms subtidal | 1.8 | 2.3 | 0.8 | 0.8 | 0.8 | 0.8 | 2.0 | 2.0 | 1.0 | 1.0 | 2.3 | 2.0 | 1.3 | 1.8 | 2.0 | 1.5 | 3.8 | 1.8 | 2.0 | 2.3 | 1.3 | 2.3 | 2.0 | 1.8 | 2.5 | 0.8 | 2.0 | 2.0 | 1.5 | 1.3 | 3.8 |
| Oligo-Mesohaline water column | | | | | | | | | | | | | | | | | | | | | | | | | | | | | | | |
| Polyhaline water column | 3.3 | 3.8 | 4.3 | 4.5 | 3.0 | 5.0 | 2.5 | 1.0 | 1.0 | 4.5 | 1.8 | 2.8 | 5.0 | 5.0 | 2.5 | 1.8 | 4.5 | 4.5 | 4.3 | 4.3 | 4.8 | 3.8 | 4.0 | 0.5 | 2.5 | 1.3 | 3.3 | 3.8 | 3.0 | 2.3 | 4.0 |
| | 2.9 | 3.4 | 1.7 | 2.1 | 1.3 | 1.8 | 2.7 | 2.3 | 1.6 | 2.6 | 2.5 | 2.5 | 3.1 | 3.2 | 2.9 | 2.7 | 3.4 | 2.3 | 2.6 | 3.3 | 2.3 | 2.9 | 2.9 | 2.1 | 2.8 | 1.7 | 3.0 | 3.1 | 2.6 | 2.4 | 4.3 |
| | 2.875 | 2.179 | | | | | | | 2.630 | | | | | | | | | | | | | | | | | | 3.075 | | | | |

## S5 Table. Trending Scenario – Zone 1

| | 1 | 2 | 3 | 4 | 5 | 6 | 7 | 8 | 9 | 10 | 11 | 12 | 13 | 14 | 15 | 16 | 17 | 18 | 19 | 20 | 21 | 22 | 23 | 24 | 25 | 26 | 27 | 28 | 29 | 30 | 31 |
|---|---|---|---|---|---|---|---|---|---|---|---|---|---|---|---|---|---|---|---|---|---|---|---|---|---|---|---|---|---|---|---|
| | 3.1 | 1.2 | 1.4 | 1.5 | 1.7 | 1.8 | 1.9 | 1.11. | 2.1 | 2.2 | 2.4 | 2.6 | 2.7 | 2.8 | 2.9 | 2.10. | 2.12 | 2.13 | 2.14 | 2.15 | 2.17 | 2.18 | 2.19 | 2.20. | 2.22 | 2.24 | 4.1 | 4.2 | 4.3 | 4.4 | 4.5 |
| | A | P | P | P | P | P | P | P | R | R | R | R | R | R | R | R | R | R | R | R | R | R | R | R | R | R | C | C | C | C | C |
| | Biodiversity | Food: Animals | Water for industrial uses | Water for aquaculture | Water for energy use | Water for navigation | Raw materials: Renewable soil materials: sand materials: sand | Raw materials: Platform | AQR: Removing harmful particles | AQR: Air-water exchange | CR: Carbon sequestration and burial | CR: Heat exchange regulation | REE: Flood water storage | REE: Peak discharge buffering | REE: Water current reduction | REE: Wave reduction | WQR: Drainage of river water | WQR: Prevention of saline intrusion. | WQR: Dissipation of tidal and river energy | WQR: Landscape maintenance | WQIR: Transport of pollutants and excess nutrients | WQIR: Reduction of excess loads coming from the catchment | Erosion and sedimentation regulation by water bodies | Erosion and sedimentation regulation by biological mediation | Prevention of establishment of harmful invasive species | Pollination | Aesthetic information | Opportunities for recreation & tourism | Inspiration for culture, art, and design | Spiritual experience | Information for cognitive development |
| Salt flats | 2.3 | 1.3 | 0.5 | 0.5 | 0.8 | 0.5 | 1.5 | 4.3 | 2.0 | 0.8 | 1.0 | 1.5 | 1.8 | 1.5 | 1.5 | 1.0 | 1.5 | 1.0 | 1.0 | 2.3 | 1.0 | 1.3 | 1.5 | 1.3 | 1.3 | 1.5 | 1.8 | 2.3 | 1.5 | 1.5 | 4.3 |
| Mangrove swamp | 5.0 | 4.3 | 2.3 | 3.5 | 1.3 | 1.3 | 2.8 | 3.8 | 4.5 | 4.3 | 5.0 | 4.0 | 5.0 | 5.0 | 5.0 | 5.0 | 2.8 | 2.8 | 2.8 | 5.0 | 2.5 | 4.3 | 4.3 | 5.0 | 3.8 | 4.5 | 4.5 | 4.5 | 4.5 | 5.0 | 5.0 |
| Shrimp pool | 2.8 | 4.5 | 1.0 | 1.8 | 1.0 | 1.0 | 2.0 | 2.0 | 1.0 | 3.3 | 2.5 | 2.8 | 2.8 | 2.8 | 2.5 | 1.5 | 2.5 | 1.5 | 1.8 | 2.3 | 1.8 | 1.8 | 2.8 | 1.0 | 1.8 | 1.0 | 2.3 | 2.3 | 1.3 | 1.3 | 4.5 |
| Sand and mudflats | 1.5 | 1.8 | 0.5 | 0.8 | 0.5 | 1.5 | 2.5 | 2.8 | 0.3 | 1.0 | 1.5 | 1.8 | 3.5 | 3.3 | 3.3 | 2.8 | 3.3 | 1.3 | 1.8 | 3.5 | 1.3 | 1.5 | 3.5 | 2.3 | 1.5 | 1.0 | 2.5 | 1.5 | 2.0 | 2.5 | 4.3 |
| Sandy bottoms subtidal | 1.5 | 1.8 | 0.5 | 0.8 | 0.5 | 0.3 | 3.8 | 1.5 | 0.5 | 0.5 | 1.5 | 1.5 | 1.0 | 1.0 | 1.5 | 1.5 | 3.8 | 1.5 | 1.5 | 1.8 | 1.0 | 1.3 | 0.5 | 1.5 | 2.3 | 0.8 | 2.0 | 1.5 | 1.8 | 1.8 | 4.0 |
| Muddy bottoms subtidal | 1.5 | 1.5 | 0.5 | 0.5 | 0.5 | 0.5 | 2.0 | 2.0 | 0.8 | 1.0 | 2.0 | 1.8 | 1.3 | 1.5 | 1.8 | 1.5 | 3.8 | 1.5 | 2.0 | 1.8 | 1.3 | 1.8 | 2.0 | 1.5 | 2.0 | 0.5 | 1.5 | 1.8 | 1.3 | 1.0 | 3.8 |
| Oligo-Mesohaline water column | 3.3 | 3.0 | 4.0 | 3.5 | 2.3 | 4.0 | 2.5 | 0.8 | 0.8 | 4.3 | 1.8 | 3.0 | 5.0 | 5.0 | 2.3 | 1.8 | 5.0 | 4.5 | 4.3 | 4.5 | 4.3 | 3.0 | 4.5 | 0.5 | 1.8 | 1.3 | 1.3 | 2.5 | 2.0 | 1.3 | 4.3 |
| Polyhaline water column | 2.5 | 2.5 | 4.0 | 3.8 | 3.0 | 5.0 | 2.5 | 0.8 | 0.8 | 4.0 | 1.8 | 2.5 | 5.0 | 5.0 | 2.3 | 1.8 | 4.5 | 4.3 | 3.8 | 4.3 | 4.3 | 3.3 | 4.0 | 0.5 | 2.0 | 1.3 | 1.3 | 2.5 | 1.8 | 2.0 | 4.0 |
| | 2.5 | 2.6 | 1.7 | 1.9 | 1.2 | 1.8 | 2.4 | 2.2 | 1.3 | 2.4 | 2.1 | 2.3 | 3.2 | 3.1 | 2.5 | 2.1 | 3.4 | 2.3 | 2.3 | 3.2 | 2.2 | 2.3 | 2.9 | 1.7 | 2.0 | 1.5 | 2.1 | 2.3 | 2.0 | 2.0 | 4.3 |
| | 2.53 | 1.96 | | | | | | | 2.37 | | | | | | | | | | | | | | | | | | 2.55 | | | | |

## S5 Table. Trending Scenario – Churute PA

| | 1 | 2 | 3 | 4 | 5 | 6 | 7 | 8 | 9 | 10 | 11 | 12 | 13 | 14 | 15 | 16 | 17 | 18 | 19 | 20 | 21 | 22 | 23 | 24 | 25 | 26 | 27 | 28 | 29 | 30 | 31 |
|---|---|---|---|---|---|---|---|---|---|---|---|---|---|---|---|---|---|---|---|---|---|---|---|---|---|---|---|---|---|---|---|
| | 3.1 | 1.2 | 1.4 | 1.5 | 1.7 | 1.8 | 1.9 | 1.11. | 2.1 | 2.2 | 2.4 | 2.6 | 2.7 | 2.8 | 2.9 | 2.10. | 2.12 | 2.13 | 2.14 | 2.15 | 2.17 | 2.18 | 2.19 | 2.20. | 2.22 | 2.24 | 4.1 | 4.2 | 4.3 | 4.4 | 4.5 |
| | A | P | P | P | P | P | P | P | R | R | R | R | R | R | R | R | R | R | R | R | R | R | R | R | R | R | C | C | C | C | C |
| | Biodiversity | Food: Animals | Water for industrial uses | Water for aquaculture | Water for energy use | Water for navigation | Raw materials: Renewable soil materials: sand materials: sand | Raw materials: Platform | AQR: Removing harmful particles | AQR: Air-water exchange | CR: Carbon sequestration and burial | CR: Heat exchange regulation | REE: Flood water storage | REE: Peak discharge buffering | REE: Water current reduction | REE: Wave reduction | WQR: Drainage of river water | WQR: Prevention of saline intrusion. | WQR: Dissipation of tidal and river energy | WQR: Landscape maintenance | WQIR: Transport of pollutants and excess nutrients | WQIR: Reduction of excess loads coming from the catchment | Erosion and sedimentation regulation by water bodies | Erosion and sedimentation regulation by biological mediation | Prevention of establishment of harmful invasive species | Pollination | Aesthetic information | Opportunities for recreation & tourism | Inspiration for culture, art, and design | Spiritual experience | Information for cognitive development |
| Salt flats | | | | | | | | | | | | | | | | | | | | | | | | | | | | | | | |
| Mangrove swamp | 4.8 | 5.0 | 2.3 | 3.8 | 1.3 | 1.3 | 2.8 | 4.3 | 5.0 | 4.3 | 4.8 | 4.3 | 5.0 | 5.0 | 5.0 | 5.0 | 3.3 | 2.8 | 3.0 | 5.0 | 3.0 | 4.8 | 4.0 | 5.0 | 4.5 | 5.0 | 4.5 | 4.8 | 4.8 | 4.8 | 5.0 |
| Shrimp pool | 3.0 | 4.5 | 1.0 | 2.3 | 1.0 | 1.0 | 2.0 | 2.0 | 1.0 | 3.5 | 2.5 | 3.0 | 2.8 | 2.8 | 2.5 | 2.0 | 2.0 | 1.5 | 1.8 | 2.5 | 2.0 | 2.3 | 2.8 | 1.3 | 2.0 | 1.0 | 2.3 | 2.5 | 2.0 | 1.8 | 4.8 |
| Sand and mudflats | 2.8 | 4.0 | 0.5 | 1.0 | 0.5 | 2.3 | 2.8 | 3.0 | 0.8 | 1.3 | 2.0 | 2.0 | 2.5 | 2.3 | 3.3 | 3.0 | 3.3 | 1.3 | 2.0 | 2.5 | 2.0 | 1.8 | 3.3 | 2.3 | 3.0 | 1.8 | 3.0 | 3.0 | 2.8 | 3.0 | 4.3 |
| Sandy bottoms subtidal | 2.0 | 2.8 | 0.5 | 1.0 | 0.5 | 0.8 | 4.3 | 1.8 | 0.5 | 0.8 | 1.8 | 1.8 | 1.0 | 1.3 | 1.8 | 1.5 | 3.8 | 1.5 | 1.5 | 2.3 | 0.8 | 1.8 | 0.8 | 1.8 | 3.5 | 1.0 | 2.0 | 2.3 | 2.0 | 2.0 | 4.5 |
| Muddy bottoms subtidal | 1.5 | 2.3 | 0.8 | 0.5 | 0.5 | 0.8 | 2.0 | 1.8 | 1.0 | 0.8 | 2.5 | 2.0 | 1.5 | 1.5 | 2.0 | 1.5 | 3.8 | 1.5 | 2.0 | 2.5 | 1.0 | 2.5 | 2.3 | 1.8 | 3.3 | 1.0 | 1.8 | 1.8 | 1.3 | 1.5 | 4.0 |
| Oligo-Mesohaline water column | 3.3 | 3.3 | 4.3 | 4.8 | 3.0 | 4.0 | 2.5 | 0.5 | 1.0 | 4.3 | 1.8 | 3.3 | 5.0 | 5.0 | 2.8 | 2.0 | 4.8 | 4.8 | 4.3 | 4.5 | 4.8 | 4.0 | 4.8 | 1.0 | 3.3 | 1.8 | 1.8 | 4.0 | 2.5 | 2.5 | 4.3 |
| Polyhaline water column | | | | | | | | | | | | | | | | | | | | | | | | | | | | | | | |
| | 2.9 | 3.6 | 1.5 | 2.2 | 1.1 | 1.7 | 2.7 | 2.2 | 1.5 | 2.5 | 2.5 | 2.7 | 3.0 | 3.0 | 2.9 | 2.5 | 3.5 | 2.2 | 2.4 | 3.2 | 2.3 | 2.8 | 3.0 | 2.2 | 3.3 | 1.9 | 2.5 | 3.0 | 2.5 | 2.6 | 4.5 |
| | 2.88 | 2.15 | | | | | | | 2.62 | | | | | | | | | | | | | | | | | | 3.03 | | | | |

## S5 Table. Trending Scenario-El Salado PA

| | 1 | 2 | 3 | 4 | 5 | 6 | 7 | 8 | 9 | 10 | 11 | 12 | 13 | 14 | 15 | 16 | 17 | 18 | 19 | 20 | 21 | 22 | 23 | 24 | 25 | 26 | 27 | 28 | 29 | 30 | 31 |
|---|---|---|---|---|---|---|---|---|---|---|---|---|---|---|---|---|---|---|---|---|---|---|---|---|---|---|---|---|---|---|---|
| | 3.1 | 1.2 | 1.4 | 1.5 | 1.7 | 1.8 | 1.9 | 1.11. | 2.1 | 2.2 | 2.4 | 2.6 | 2.7 | 2.8 | 2.9 | 2.10. | 2.12 | 2.13 | 2.14 | 2.15 | 2.17 | 2.18 | 2.19 | 2.20. | 2.22 | 2.24 | 4.1 | 4.2 | 4.3 | 4.4 | 4.5 |
| | A | P | P | P | P | P | P | P | R | R | R | R | R | R | R | R | R | R | R | R | R | R | R | R | R | R | C | C | C | C | C |
| | Biodiversity | Food: Animals | Water for industrial uses | Water for aquaculture | Water for energy use | Water for navigation | Raw materials: Renewable soil materials: sand materials: sand | Raw materials: Platform | AQR: Removing harmful particles | AQR: Air-water exchange | CR: Carbon sequestration and burial | CR: Heat exchange regulation | REE: Flood water storage | REE: Peak discharge buffering | REE: Water current reduction | REE: Wave reduction | WQR: Drainage of river water | WQR: Prevention of saline intrusion. | WQR: Dissipation of tidal and river energy | WQR: Landscape maintenance | WQIR: Transport of pollutants and excess nutrients | WQIR: Reduction of excess loads coming from the catchment | Erosion and sedimentation regulation by water bodies | Erosion and sedimentation regulation by biological mediation | Prevention of establishment of harmful invasive species | Pollination | Aesthetic information | Opportunities for recreation & tourism | Inspiration for culture, art, and design | Spiritual experience | Information for cognitive development |
| Salt flats | 2.3 | 1.3 | 0.3 | 0.5 | 0.8 | 0.5 | 1.5 | 4.5 | 2.0 | 0.8 | 1.3 | 1.8 | 2.0 | 1.8 | 1.5 | 1.3 | 1.5 | 1.3 | 1.0 | 2.8 | 1.0 | 1.0 | 1.5 | 1.3 | 1.8 | 1.5 | 2.0 | 2.3 | 1.8 | 1.8 | 4.0 |
| Mangrove swamp | 4.5 | 4.5 | 2.3 | 3.3 | 1.0 | 1.3 | 2.8 | 4.3 | 4.3 | 4.0 | 4.5 | 4.3 | 4.5 | 4.5 | 4.5 | 5.0 | 3.0 | 2.8 | 2.3 | 5.0 | 2.3 | 4.8 | 4.0 | 4.8 | 4.3 | 5.0 | 4.0 | 4.3 | 4.0 | 4.5 | 4.8 |
| Shrimp pool | 3.0 | 4.0 | 1.0 | 2.0 | 1.0 | 1.3 | 2.0 | 2.0 | 1.0 | 3.5 | 2.5 | 2.8 | 2.8 | 2.5 | 2.3 | 1.5 | 2.5 | 1.5 | 1.8 | 2.3 | 1.5 | 1.8 | 2.8 | 1.3 | 1.8 | 1.3 | 2.5 | 2.8 | 2.0 | 1.3 | 4.3 |
| Sand and mudflats | 1.5 | 2.0 | 0.8 | 0.8 | 0.5 | 2.0 | 2.5 | 2.5 | 0.5 | 1.3 | 1.8 | 2.0 | 2.5 | 3.3 | 3.3 | 2.5 | 3.3 | 1.3 | 2.3 | 3.3 | 1.3 | 1.8 | 3.3 | 2.8 | 2.3 | 1.0 | 2.8 | 1.5 | 2.8 | 2.8 | 4.3 |
| Sandy bottoms subtidal | | | | | | | | | | | | | | | | | | | | | | | | | | | | | | | |
| Muddy bottoms subtidal | 2.0 | 1.5 | 0.8 | 0.8 | 0.8 | 0.8 | 2.0 | 2.0 | 0.8 | 0.8 | 2.0 | 2.0 | 1.5 | 1.5 | 2.0 | 1.5 | 3.8 | 1.5 | 2.0 | 2.5 | 1.3 | 2.5 | 2.3 | 1.5 | 2.5 | 0.8 | 2.0 | 2.0 | 1.3 | 1.5 | 3.5 |
| Oligo-Mesohaline water column | | | | | | | | | | | | | | | | | | | | | | | | | | | | | | | |
| Polyhaline water column | 2.8 | 2.5 | 4.0 | 3.8 | 3.8 | 4.8 | 2.5 | 0.8 | 0.8 | 4.0 | 1.8 | 2.8 | 5.0 | 5.0 | 2.3 | 1.5 | 4.8 | 4.0 | 3.5 | 4.3 | 4.0 | 3.8 | 4.0 | 0.5 | 2.3 | 1.3 | 1.5 | 2.0 | 2.8 | 2.3 | 4.0 |
| | 2.7 | 2.6 | 1.5 | 1.8 | 1.3 | 1.8 | 2.2 | 2.7 | 1.5 | 2.4 | 2.3 | 2.6 | 3.0 | 3.1 | 2.6 | 2.2 | 3.1 | 2.0 | 2.1 | 3.3 | 1.9 | 2.6 | 3.0 | 2.0 | 2.5 | 1.8 | 2.5 | 2.5 | 2.4 | 2.3 | 4.1 |
| | 2.67 | 1.98 | | | | | | | 2.45 | | | | | | | | | | | | | | | | | | 2.76 | | | | |

## S5 Table. Trending Scenario-El Morro PA

| | 1 | 2 | 3 | 4 | 5 | 6 | 7 | 8 | 9 | 10 | 11 | 12 | 13 | 14 | 15 | 16 | 17 | 18 | 19 | 20 | 21 | 22 | 23 | 24 | 25 | 26 | 27 | 28 | 29 | 30 | 31 |
|---|---|---|---|---|---|---|---|---|---|---|---|---|---|---|---|---|---|---|---|---|---|---|---|---|---|---|---|---|---|---|---|
| | 3.1 | 1.2 | 1.4 | 1.5 | 1.7 | 1.8 | 1.9 | 1.11. | 2.1 | 2.2 | 2.4 | 2.6 | 2.7 | 2.8 | 2.9 | 2.10. | 2.12 | 2.13 | 2.14 | 2.15 | 2.17 | 2.18 | 2.19 | 2.20. | 2.22 | 2.24 | 4.1 | 4.2 | 4.3 | 4.4 | 4.5 |
| | A | P | P | P | P | P | P | P | R | R | R | R | R | R | R | R | R | R | R | R | R | R | R | R | R | R | C | C | C | C | C |
| | Biodiversity | Food: Animals | Water for industrial uses | Water for aquaculture | Water for energy use | Water for navigation | Raw materials: Renewable soil materials: | Raw materials: Platform | AQR: Removing harmful particles | AQR: Air-water exchange | CR: Carbon sequestration and burial | CR: Heat exchange regulation | REE: Flood water storage | REE: Peak discharge buffering | REE: Water current reduction | REE: Wave reduction | WQR: Drainage of river water | WQR: Prevention of saline intrusion. | WQR: Dissipation of tidal and river energy | WQR: Landscape maintenance | WQIR: Transport of pollutants and excess nutrients | WQIR: Reduction of excess loads coming from the catchment | Erosion and sedimentation regulation by water bodies | Erosion and sedimentation regulation by biological mediation | Prevention of establishment of harmful invasive species | Pollination | Aesthetic information | Opportunities for recreation & tourism | Inspiration for culture, art, and design | Spiritual experience | Information for cognitive development |
| Salt flats | | | | | | | | | | | | | | | | | | | | | | | | | | | | | | | |
| Mangrove swamp | 4.5 | 4.0 | 2.8 | 3.0 | 1.3 | 1.3 | 2.8 | 4.3 | 5.0 | 4.3 | 5.0 | 4.3 | 4.5 | 4.8 | 5.0 | 4.5 | 2.8 | 2.5 | 3.0 | 4.8 | 2.5 | 4.3 | 4.0 | 4.8 | 4.8 | 5.0 | 4.5 | 5.0 | 4.5 | 5.0 | 4.8 |
| Shrimp pool | 3.0 | 4.5 | 1.0 | 2.3 | 1.0 | 1.0 | 2.0 | 2.0 | 1.0 | 3.0 | 2.3 | 2.8 | 2.8 | 2.8 | 2.5 | 2.5 | 2.0 | 1.5 | 2.3 | 2.3 | 2.0 | 2.3 | 2.8 | 1.0 | 1.8 | 1.0 | 2.0 | 2.5 | 1.5 | 1.3 | 4.5 |
| Sand and mudflats | 2.5 | 2.5 | 0.8 | 1.0 | 0.8 | 2.0 | 2.8 | 2.8 | 1.0 | 1.5 | 1.8 | 1.8 | 3.3 | 3.3 | 3.3 | 3.3 | 3.3 | 1.5 | 2.0 | 3.5 | 1.5 | 2.3 | 3.3 | 2.8 | 2.3 | 1.3 | 2.8 | 3.0 | 2.3 | 2.5 | 4.5 |
| Sandy bottoms subtidal | 1.8 | 2.0 | 0.5 | 1.0 | 0.8 | 0.5 | 4.0 | 1.5 | 0.5 | 0.8 | 1.8 | 1.8 | 1.3 | 1.3 | 1.8 | 1.5 | 3.8 | 1.5 | 1.8 | 2.0 | 1.5 | 2.0 | 0.5 | 1.5 | 2.5 | 1.0 | 1.8 | 1.8 | 1.8 | 1.5 | 4.3 |
| Muddy bottoms subtidal | 1.8 | 1.8 | 0.8 | 0.8 | 0.8 | 0.5 | 2.0 | 2.0 | 1.0 | 1.0 | 2.0 | 2.0 | 1.3 | 1.5 | 1.8 | 1.5 | 3.8 | 1.5 | 2.0 | 2.3 | 1.3 | 2.3 | 2.0 | 1.5 | 2.0 | 0.8 | 1.8 | 1.8 | 1.5 | 1.3 | 3.8 |
| Oligo-Mesohaline water column | | | | | | | | | | | | | | | | | | | | | | | | | | | | | | | |
| Polyhaline water column | 2.8 | 3.3 | 4.3 | 4.0 | 3.0 | 4.8 | 2.5 | 0.8 | 1.0 | 4.3 | 1.8 | 2.5 | 4.8 | 5.0 | 2.5 | 1.5 | 4.5 | 4.3 | 4.3 | 4.3 | 4.0 | 2.8 | 4.0 | 0.5 | 2.0 | 1.3 | 2.8 | 3.3 | 3.0 | 2.0 | 4.0 |
| | 2.7 | 3.0 | 1.7 | 2.0 | 1.3 | 1.7 | 2.7 | 2.2 | 1.6 | 2.5 | 2.4 | 2.5 | 3.0 | 3.1 | 2.8 | 2.5 | 3.3 | 2.1 | 2.5 | 3.2 | 2.1 | 2.6 | 2.8 | 2.0 | 2.5 | 1.7 | 2.6 | 2.9 | 2.4 | 2.3 | 4.3 |
| | 2.71 | 2.07 | | | | | | | 2.51 | | | | | | | | | | | | | | | | | | 2.88 | | | | |

## S5 Table. Exploratory Scenario-Zone 1

| | 1 | 2 | 3 | 4 | 5 | 6 | 7 | 8 | 9 | 10 | 11 | 12 | 13 | 14 | 15 | 16 | 17 | 18 | 19 | 20 | 21 | 22 | 23 | 24 | 25 | 26 | 27 | 28 | 29 | 30 | 31 |
|---|---|---|---|---|---|---|---|---|---|---|---|---|---|---|---|---|---|---|---|---|---|---|---|---|---|---|---|---|---|---|---|
| | 3.1 | 1.2 | 1.4 | 1.5 | 1.7 | 1.8 | 1.9 | 1.11. | 2.1 | 2.2 | 2.4 | 2.6 | 2.7 | 2.8 | 2.9 | 2.10. | 2.12 | 2.13 | 2.14 | 2.15 | 2.17 | 2.18 | 2.19 | 2.20. | 2.22 | 2.24 | 4.1 | 4.2 | 4.3 | 4.4 | 4.5 |
| | A | P | P | P | P | P | P | P | R | R | R | R | R | R | R | R | R | R | R | R | R | R | R | R | R | R | C | C | C | C | C |
| | Biodiversity | Food: Animals | Water for industrial uses | Water for aquaculture | Water for energy use | Water for navigation | Raw materials: Renewable soil materials: sand materials: sand | Raw materials: Platform | AQR: Removing harmful particles | AQR: Air-water exchange | CR: Carbon sequestration and burial | CR: Heat exchange regulation | REE: Flood water storage | REE: Peak discharge buffering | REE: Water current reduction | REE: Wave reduction | WQR: Drainage of river water | WQR: Prevention of saline intrusion. | WQR: Dissipation of tidal and river energy | WQR: Landscape maintenance | WQIR: Transport of pollutants and excess nutrients | WQIR: Reduction of excess loads coming from the catchment | Erosion and sedimentation regulation by water bodies | Erosion and sedimentation regulation by biological mediation | Prevention of establishment of harmful invasive species | Pollination | Aesthetic information | Opportunities for recreation & tourism | Inspiration for culture, art, and design | Spiritual experience | Information for cognitive development |
| Salt flats | 2.8 | 1.3 | 0.5 | 0.5 | 0.8 | 0.5 | 1.3 | 4.0 | 1.8 | 0.5 | 1.0 | 1.8 | 1.5 | 1.5 | 1.5 | 1.0 | 1.0 | 1.0 | 0.8 | 1.3 | 1.0 | 1.3 | 1.0 | 1.3 | 1.3 | 1.0 | 1.8 | 1.8 | 1.0 | 1.3 | 3.8 |
| Mangrove swamp | 3.5 | 3.3 | 2.3 | 3.0 | 1.3 | 1.3 | 2.8 | 4.0 | 3.8 | 3.8 | 3.8 | 3.8 | 4.3 | 4.0 | 4.3 | 4.5 | 2.5 | 2.3 | 2.8 | 4.0 | 2.5 | 3.0 | 3.5 | 4.0 | 3.5 | 3.5 | 3.8 | 3.8 | 3.8 | 4.3 | 4.5 |
| Shrimp pool | 2.0 | 5.0 | 1.0 | 1.8 | 1.0 | 1.0 | 1.8 | 2.0 | 0.8 | 3.3 | 1.8 | 2.8 | 2.8 | 2.8 | 2.5 | 1.5 | 2.0 | 1.3 | 1.8 | 1.8 | 1.8 | 1.8 | 3.3 | 1.0 | 1.5 | 1.0 | 1.3 | 1.8 | 1.5 | 1.3 | 4.8 |
| Sand and mudflats | 1.5 | 1.5 | 0.3 | 0.8 | 0.5 | 1.3 | 2.5 | 2.5 | 0.5 | 0.8 | 1.5 | 1.8 | 3.5 | 3.3 | 3.3 | 2.3 | 3.3 | 1.3 | 1.8 | 3.0 | 1.3 | 1.5 | 3.0 | 2.0 | 1.5 | 1.0 | 2.0 | 1.3 | 1.5 | 1.8 | 3.8 |
| Sandy bottoms subtidal | 1.8 | 1.0 | 0.5 | 0.5 | 0.5 | 0.5 | 3.8 | 1.5 | 0.3 | 0.5 | 1.0 | 1.5 | 1.3 | 1.0 | 1.3 | 1.5 | 3.8 | 1.5 | 1.5 | 1.8 | 1.0 | 1.5 | 0.5 | 1.5 | 2.0 | 0.5 | 1.3 | 1.3 | 1.3 | 1.8 | 4.3 |
| Muddy bottoms subtidal | 1.5 | 1.3 | 0.5 | 0.5 | 0.5 | 0.5 | 1.8 | 1.8 | 0.8 | 1.0 | 1.5 | 1.8 | 1.3 | 1.5 | 1.8 | 1.5 | 3.8 | 1.3 | 2.0 | 1.8 | 1.3 | 1.3 | 2.0 | 1.5 | 1.8 | 0.8 | 1.3 | 1.3 | 1.3 | 1.3 | 3.8 |
| Oligo-Mesohaline water column | 3.5 | 2.0 | 3.8 | 3.3 | 2.3 | 3.5 | 2.5 | 0.5 | 0.8 | 4.0 | 1.5 | 2.8 | 4.5 | 5.0 | 2.3 | 1.8 | 4.8 | 4.3 | 4.0 | 4.5 | 4.0 | 2.8 | 4.3 | 0.5 | 1.8 | 1.3 | 1.3 | 2.3 | 2.0 | 1.3 | 4.0 |
| Polyhaline water column | 2.3 | 2.0 | 3.8 | 3.5 | 3.0 | 4.0 | 2.5 | 0.8 | 0.5 | 3.8 | 1.5 | 2.3 | 4.5 | 4.8 | 2.3 | 1.8 | 4.3 | 4.0 | 3.8 | 4.3 | 4.0 | 2.5 | 4.0 | 0.3 | 2.0 | 1.3 | 1.3 | 2.5 | 1.8 | 2.3 | 4.0 |
| | 2.3 | 2.2 | 1.6 | 1.7 | 1.2 | 1.6 | 2.3 | 2.1 | 1.1 | 2.2 | 1.7 | 2.3 | 2.9 | 3.0 | 2.4 | 2.0 | 3.2 | 2.1 | 2.3 | 2.8 | 2.1 | 1.9 | 2.7 | 1.5 | 1.9 | 1.3 | 1.7 | 2.0 | 1.8 | 1.9 | 4.1 |
| | 2.34 | 1.81 | | | | | | | 2.18 | | | | | | | | | | | | | | | | | | 2.28 | | | | |

## S5 Table. Exploratory Scenario – Churute AP

| | 1 | 2 | 3 | 4 | 5 | 6 | 7 | 8 | 9 | 10 | 11 | 12 | 13 | 14 | 15 | 16 | 17 | 18 | 19 | 20 | 21 | 22 | 23 | 24 | 25 | 26 | 27 | 28 | 29 | 30 | 31 |
|---|---|---|---|---|---|---|---|---|---|---|---|---|---|---|---|---|---|---|---|---|---|---|---|---|---|---|---|---|---|---|---|
| | 3.1 | 1.2 | 1.4 | 1.5 | 1.7 | 1.8 | 1.9 | 1.11. | 2.1 | 2.2 | 2.4 | 2.6 | 2.7 | 2.8 | 2.9 | 2.10. | 2.12 | 2.13 | 2.14 | 2.15 | 2.17 | 2.18 | 2.19 | 2.20. | 2.22 | 2.24 | 4.1 | 4.2 | 4.3 | 4.4 | 4.5 |
| | A | P | P | P | P | P | P | P | R | R | R | R | R | R | R | R | R | R | R | R | R | R | R | R | R | R | C | C | C | C | C |
| | Biodiversity | Food: Animals | Water for industrial uses | Water for aquaculture | Water for energy use | Water for navigation | Raw materials: Renewable soil materials: sand materials: sand | Raw materials: Platform | AQR: Removing harmful particles | AQR: Air-water exchange | CR: Carbon sequestration and burial | CR: Heat exchange regulation | REE: Flood water storage | REE: Peak discharge buffering | REE: Water current reduction | REE: Wave reduction | WQR: Drainage of river water | WQR: Prevention of saline intrusion. | WQR: Dissipation of tidal and river energy | WQR: Landscape maintenance | WQIR: Transport of pollutants and excess nutrients | WQIR: Reduction of excess loads coming from the catchment | Erosion and sedimentation regulation by water bodies | Erosion and sedimentation regulation by biological mediation | Prevention of establishment of harmful invasive species | Pollination | Aesthetic information | Opportunities for recreation & tourism | Inspiration for culture, art, and design | Spiritual experience | Information for cognitive development |
| Salt flats | | | | | | | | | | | | | | | | | | | | | | | | | | | | | | | |
| Mangrove swamp | 4.5 | 3.8 | 2.3 | 3.0 | 1.3 | 1.3 | 2.8 | 4.0 | 4.5 | 4.3 | 4.5 | 4.0 | 4.5 | 5.0 | 4.5 | 4.5 | 3.3 | 2.8 | 3.0 | 4.8 | 2.5 | 4.0 | 3.5 | 4.3 | 4.5 | 4.3 | 4.3 | 4.3 | 4.3 | 4.5 | 4.5 |
| Shrimp pool | 3.0 | 4.5 | 0.8 | 1.8 | 1.3 | 1.0 | 2.0 | 2.0 | 1.0 | 3.5 | 2.5 | 3.0 | 2.5 | 2.5 | 2.5 | 2.0 | 1.8 | 1.3 | 1.8 | 2.5 | 2.3 | 2.3 | 3.3 | 1.3 | 2.3 | 1.0 | 2.3 | 2.5 | 1.8 | 1.8 | 5.0 |
| Sand and mudflats | 2.8 | 3.3 | 0.5 | 0.8 | 0.5 | 1.5 | 2.8 | 2.8 | 0.8 | 1.0 | 1.8 | 2.0 | 2.5 | 2.3 | 3.3 | 3.0 | 3.3 | 1.3 | 2.0 | 2.5 | 1.8 | 2.0 | 3.0 | 1.8 | 2.5 | 1.0 | 2.5 | 2.5 | 2.3 | 2.3 | 3.8 |
| Sandy bottoms subtidal | 2.0 | 2.3 | 0.5 | 1.0 | 0.5 | 0.8 | 4.0 | 1.8 | 0.8 | 0.8 | 1.5 | 1.5 | 1.0 | 1.3 | 1.5 | 1.3 | 3.8 | 1.5 | 1.8 | 2.3 | 0.8 | 2.0 | 0.8 | 1.8 | 2.5 | 1.0 | 1.8 | 2.3 | 2.0 | 1.8 | 4.0 |
| Muddy bottoms subtidal | 1.5 | 1.8 | 0.5 | 0.8 | 0.5 | 0.5 | 2.0 | 2.0 | 1.0 | 1.0 | 2.3 | 2.0 | 1.3 | 1.5 | 2.0 | 1.5 | 3.8 | 1.3 | 2.0 | 2.5 | 0.8 | 2.3 | 2.3 | 1.8 | 2.8 | 1.0 | 2.0 | 1.8 | 1.3 | 1.3 | 3.8 |
| Oligo-Mesohaline water column | 3.3 | 3.3 | 4.3 | 4.3 | 3.0 | 4.0 | 2.5 | 0.5 | 1.0 | 4.3 | 1.8 | 3.0 | 4.8 | 4.8 | 2.8 | 2.0 | 4.8 | 4.8 | 4.3 | 4.8 | 4.8 | 4.0 | 4.5 | 1.0 | 2.8 | 1.8 | 2.0 | 3.3 | 2.3 | 2.5 | 4.3 |
| Polyhaline water column | | | | | | | | | | | | | | | | | | | | | | | | | | | | | | | |
| | 2.8 | 3.1 | 1.5 | 1.9 | 1.2 | 1.5 | 2.7 | 2.2 | 1.5 | 2.5 | 2.4 | 2.6 | 2.8 | 2.9 | 2.8 | 2.4 | 3.4 | 2.1 | 2.5 | 3.2 | 2.1 | 2.8 | 2.9 | 2.0 | 2.9 | 1.7 | 2.5 | 2.8 | 2.3 | 2.3 | 4.2 |
| | 2.83 | 2.00 | | | | | | | 2.51 | | | | | | | | | | | | | | | | | | 2.81 | | | | |

## S5 Table. Exploratory Scenario – El Salado PA

| | 1 | 2 | 3 | 4 | 5 | 6 | 7 | 8 | 9 | 10 | 11 | 12 | 13 | 14 | 15 | 16 | 17 | 18 | 19 | 20 | 21 | 22 | 23 | 24 | 25 | 26 | 27 | 28 | 29 | 30 | 31 |
|---|---|---|---|---|---|---|---|---|---|---|---|---|---|---|---|---|---|---|---|---|---|---|---|---|---|---|---|---|---|---|---|
| | 3.1 | 1.2 | 1.4 | 1.5 | 1.7 | 1.8 | 1.9 | 1.11. | 2.1 | 2.2 | 2.4 | 2.6 | 2.7 | 2.8 | 2.9 | 2.10. | 2.12 | 2.13 | 2.14 | 2.15 | 2.17 | 2.18 | 2.19 | 2.20. | 2.22 | 2.24 | 4.1 | 4.2 | 4.3 | 4.4 | 4.5 |
| | A | P | P | P | P | P | P | P | R | R | R | R | R | R | R | R | R | R | R | R | R | R | R | R | R | R | C | C | C | C | C |
| | Biodiversity | Food: Animals | Water for industrial uses | Water for aquaculture | Water for energy use | Water for navigation | Raw materials: Renewable soil materials: sand materials: sand | Raw materials: Platform | AQR: Removing harmful particles | AQR: Air-water exchange | CR: Carbon sequestration and burial | CR: Heat exchange regulation | REE: Flood water storage | REE: Peak discharge buffering | REE: Water current reduction | REE: Wave reduction | WQR: Drainage of river water | WQR: Prevention of saline intrusion. | WQR: Dissipation of tidal and river energy | WQR: Landscape maintenance | WQIR: Transport of pollutants and excess nutrients | WQIR: Reduction of excess loads coming from the catchment | Erosion and sedimentation regulation by water bodies | Erosion and sedimentation regulation by biological mediation | Prevention of establishment of harmful invasive species | Pollination | Aesthetic information | Opportunities for recreation & tourism | Inspiration for culture, art, and design | Spiritual experience | Information for cognitive development |
| Salt flats | 2.8 | 1.5 | 0.5 | 0.5 | 0.8 | 0.5 | 1.5 | 4.5 | 2.0 | 0.8 | 1.3 | 1.8 | 1.8 | 1.8 | 1.5 | 1.3 | 1.5 | 1.3 | 1.0 | 1.5 | 1.0 | 1.3 | 1.5 | 1.3 | 1.3 | 1.3 | 2.0 | 1.8 | 1.0 | 1.5 | 3.8 |
| Mangrove swamp | 3.8 | 3.3 | 2.0 | 3.5 | 1.0 | 1.0 | 2.5 | 4.8 | 3.5 | 4.0 | 4.0 | 3.8 | 4.0 | 3.8 | 4.0 | 3.8 | 2.5 | 2.5 | 2.5 | 3.8 | 2.5 | 4.0 | 4.3 | 3.8 | 3.3 | 4.5 | 3.3 | 3.5 | 3.0 | 3.8 | 4.3 |
| Shrimp pool | 3.0 | 4.8 | 1.0 | 2.0 | 1.0 | 1.3 | 2.0 | 2.0 | 1.0 | 3.3 | 2.0 | 2.8 | 2.8 | 2.5 | 2.3 | 1.5 | 2.0 | 1.5 | 1.8 | 1.8 | 1.5 | 2.5 | 2.8 | 1.3 | 1.8 | 1.3 | 2.0 | 2.0 | 2.0 | 1.5 | 4.3 |
| Sand and mudflats | 1.5 | 1.8 | 0.8 | 1.0 | 0.8 | 1.5 | 2.3 | 2.8 | 0.8 | 1.3 | 1.8 | 2.0 | 2.8 | 3.3 | 3.3 | 2.3 | 3.3 | 1.5 | 2.0 | 3.3 | 1.3 | 1.8 | 3.3 | 2.0 | 2.3 | 1.0 | 2.3 | 1.5 | 1.5 | 2.0 | 3.8 |
| Sandy bottoms subtidal | | | | | | | | | | | | | | | | | | | | | | | | | | | | | | | |
| Muddy bottoms subtidal | 2.3 | 1.8 | 0.8 | 0.8 | 0.8 | 0.8 | 2.0 | 2.0 | 0.8 | 1.0 | 2.0 | 2.0 | 1.5 | 1.5 | 2.0 | 1.5 | 3.8 | 1.5 | 2.0 | 2.3 | 1.3 | 2.3 | 2.3 | 1.5 | 2.5 | 0.8 | 1.3 | 1.8 | 1.3 | 1.3 | 3.5 |
| Oligo-Mesohaline water column | | | | | | | | | | | | | | | | | | | | | | | | | | | | | | | |
| Polyhaline water column | 2.5 | 2.5 | 3.3 | 3.5 | 3.5 | 4.5 | 2.3 | 0.8 | 0.5 | 3.5 | 1.8 | 2.8 | 4.5 | 4.8 | 2.3 | 1.5 | 5.0 | 3.8 | 3.8 | 4.3 | 3.8 | 3.8 | 4.3 | 0.5 | 2.5 | 1.3 | 1.5 | 2.0 | 2.0 | 2.3 | 4.0 |
| | 2.6 | 2.6 | 1.4 | 1.9 | 1.3 | 1.6 | 2.1 | 2.8 | 1.4 | 2.3 | 2.1 | 2.5 | 2.9 | 2.9 | 2.5 | 2.0 | 3.0 | 2.0 | 2.2 | 2.8 | 1.9 | 2.6 | 3.0 | 1.7 | 2.3 | 1.7 | 2.0 | 2.1 | 1.8 | 2.0 | 3.9 |
| | 2.63 | 1.94 | | | | | | | 2.32 | | | | | | | | | | | | | | | | | | 2.38 | | | | |

## S5 Table. Exploratory Scenario - El Morro PA

| | 1 | 2 | 3 | 4 | 5 | 6 | 7 | 8 | 9 | 10 | 11 | 12 | 13 | 14 | 15 | 16 | 17 | 18 | 19 | 20 | 21 | 22 | 23 | 24 | 25 | 26 | 27 | 28 | 29 | 30 | 31 |
|---|---|---|---|---|---|---|---|---|---|---|---|---|---|---|---|---|---|---|---|---|---|---|---|---|---|---|---|---|---|---|---|
| | 3.1 | 1.2 | 1.4 | 1.5 | 1.7 | 1.8 | 1.9 | 1.11. | 2.1 | 2.2 | 2.4 | 2.6 | 2.7 | 2.8 | 2.9 | 2.10. | 2.12 | 2.13 | 2.14 | 2.15 | 2.17 | 2.18 | 2.19 | 2.20. | 2.22 | 2.24 | 4.1 | 4.2 | 4.3 | 4.4 | 4.5 |
| | A | P | P | P | P | P | P | P | R | R | R | R | R | R | R | R | R | R | R | R | R | R | R | R | R | R | C | C | C | C | C |
| | Biodiversity | Food: Animals | Water for industrial uses | Water for aquaculture | Water for energy use | Water for navigation | Raw materials: Renewable soil materials: sand materials: sand | Raw materials: Platform | AQR: Removing harmful particles | AQR: Air-water exchange | CR: Carbon sequestration and burial | CR: Heat exchange regulation | REE: Flood water storage | REE: Peak discharge buffering | REE: Water current reduction | REE: Wave reduction | WQR: Drainage of river water | WQR: Prevention of saline intrusion. | WQR: Dissipation of tidal and river energy | WQR: Landscape maintenance | WQIR: Transport of pollutants and excess nutrients | WQIR: Reduction of excess loads coming from the catchment | Erosion and sedimentation regulation by water bodies | Erosion and sedimentation regulation by biological mediation | Prevention of establishment of harmful invasive species | Pollination | Aesthetic information | Opportunities for recreation & tourism | Inspiration for culture, art, and design | Spiritual experience | Information for cognitive development |
| Salt flats | | | | | | | | | | | | | | | | | | | | | | | | | | | | | | | |
| Mangrove swamp | 3.8 | 3.5 | 2.5 | 2.8 | 1.0 | 1.0 | 2.5 | 4.0 | 4.0 | 3.8 | 4.3 | 3.8 | 4.0 | 4.3 | 4.5 | 4.3 | 2.8 | 2.8 | 2.8 | 4.0 | 2.5 | 4.0 | 3.3 | 4.0 | 4.0 | 4.3 | 4.3 | 4.3 | 4.3 | 4.0 | 4.5 |
| Shrimp pool | 2.8 | 4.8 | 0.8 | 2.0 | 1.0 | 1.0 | 2.0 | 2.0 | 0.8 | 3.3 | 2.5 | 2.8 | 2.5 | 2.3 | 2.5 | 2.5 | 2.0 | 1.5 | 2.0 | 2.0 | 1.8 | 2.3 | 3.3 | 1.0 | 1.8 | 0.8 | 1.5 | 2.3 | 1.5 | 1.3 | 4.3 |
| Sand and mudflats | 1.8 | 2.5 | 0.8 | 1.0 | 0.8 | 2.0 | 2.8 | 2.5 | 1.0 | 1.3 | 1.8 | 1.8 | 3.0 | 3.3 | 3.5 | 2.8 | 3.3 | 1.3 | 2.3 | 3.5 | 1.5 | 2.0 | 3.0 | 2.0 | 1.8 | 1.3 | 2.0 | 2.5 | 2.3 | 2.0 | 4.5 |
| Sandy bottoms subtidal | 1.5 | 1.8 | 0.8 | 1.0 | 0.8 | 0.5 | 3.8 | 1.5 | 0.8 | 1.0 | 1.8 | 1.8 | 1.3 | 1.0 | 1.8 | 1.5 | 3.8 | 1.3 | 1.5 | 2.0 | 1.5 | 2.0 | 0.5 | 1.5 | 2.5 | 0.8 | 1.3 | 1.8 | 1.8 | 1.5 | 4.3 |
| Muddy bottoms subtidal | 1.8 | 1.8 | 0.8 | 0.8 | 0.5 | 0.8 | 2.0 | 2.0 | 1.0 | 1.0 | 2.0 | 2.0 | 1.3 | 1.5 | 1.8 | 1.5 | 3.8 | 1.5 | 2.0 | 2.3 | 1.3 | 2.3 | 2.0 | 1.8 | 1.8 | 0.8 | 1.3 | 1.8 | 1.3 | 1.3 | 3.5 |
| Oligo-Mesohaline water column | | | | | | | | | | | | | | | | | | | | | | | | | | | | | | | |
| Polyhaline water column | 3.0 | 3.0 | 4.0 | 4.0 | 3.0 | 5.0 | 2.3 | 0.8 | 1.0 | 4.3 | 1.8 | 2.3 | 4.5 | 5.0 | 2.3 | 1.8 | 4.5 | 4.5 | 4.0 | 4.3 | 4.3 | 2.5 | 4.0 | 0.5 | 2.0 | 1.3 | 2.8 | 3.0 | 3.0 | 2.3 | 4.0 |
| | 2.4 | 2.9 | 1.6 | 1.9 | 1.2 | 1.7 | 2.5 | 2.1 | 1.4 | 2.4 | 2.3 | 2.4 | 2.8 | 2.9 | 2.7 | 2.4 | 3.3 | 2.1 | 2.4 | 3.0 | 2.1 | 2.5 | 2.7 | 1.8 | 2.3 | 1.5 | 2.2 | 2.6 | 2.3 | 2.0 | 4.2 |
| | 2.417 | 1.988 | | | | | | | 2.389 | | | | | | | | | | | | | | | | | | 2.658 | | | | |
