# Supplementary material for: Assessment of ecosystem services of an urbanized tropical estuary with a focus on habitats and scenarios
Source: PLoS One. 2018 Oct 5;13(10):e0203927. doi: 10.1371/journal.pone.0203927 (PMC6173385; doi:10.1371/journal.pone.0203927)
Supplement: S1 Fig — (PDF) [file pone.0203927.s008.pdf]

SUPPORTING INFORMATION

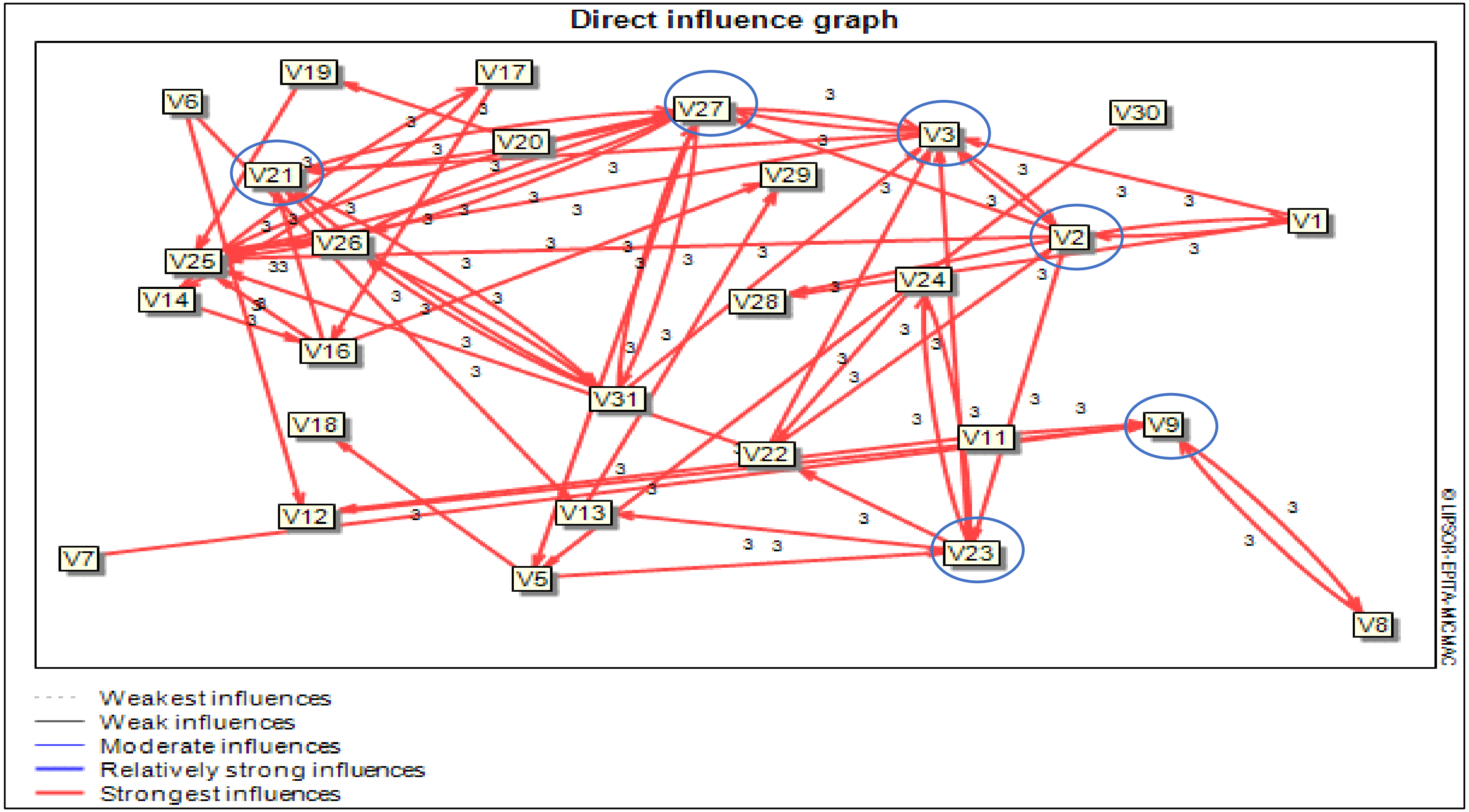

S1 Fig. CAUSAL ANALYSIS : This graph was carried out with MICMAC software. The key variables are marked with a blue circle.
